# Seasonal and temporal patterns of rainfall shape arthropod community composition and multi-trophic interactions in an arid environment

**DOI:** 10.1038/s41598-022-07716-0

**Published:** 2022-03-08

**Authors:** Christina Fischer, Roland Gerstmeier, Thomas C. Wagner

**Affiliations:** 1 Faunistics and Wildlife Conservation, Department of Agriculture, Ecotrophology, and Landscape Development, Anhalt University of Applied Sciences, Strenzfelder Allee 28, 06406 Bernburg, Germany; 2 Restoration Ecology, School of Life Sciences, Technische Universität München, Freising, Germany; 3 Entomology, Chair of Zoology, School of Life Sciences, Technische Universität München, Freising, Germany

**Keywords:** Biodiversity, Climate-change ecology, Community ecology, Ecological networks, Ecosystem ecology, Grassland ecology, Population dynamics, Tropical ecology, Biodiversity, Climate-change ecology, Community ecology, Conservation biology, Ecological networks, Ecosystem ecology, Population dynamics, Tropical ecology

## Abstract

In arid and semi-arid ecosystems, rainfall and rainfall temporal distribution shape species communities and multi-trophic interactions. Whereas the relationship between climate change-induced decline of precipitation and plants is well know, there is little knowledge of these relationships with consumers, such as arthropods of different trophic levels. In a 6-year period we studied precipitation effects and microhabitat conditions on multi-trophic interactions of ground-dwelling arthropods in an arid savannah. We analysed the effects of seasonal rainfall, plant cover and soil texture on community composition and activity density of arthropods of different trophic levels and investigated the critical window of vegetation and occurrence arthropods in relation to rainfall. Our result show, that arthropod community composition was determined by seasonal rainfall and plant cover. Soil texture did not explain arthropod response sufficiently. Especially detritivorous arthropods were strongly affected by precipitation and can therefore serve as indicators of droughts. Further, multi-trophic interactions can better be explained by short-term rainfall pulses, rather than by seasonal patterns, with a window of seven days being most suitable to explain the influence of rainfall. Plant cover responded immediately after the rainfall, followed by herbivorous and predatory arthropods, and with a lag of 23 days omnivorous arthropods. This highlights the importance of short-term rain pulses for multi-trophic interactions among arthropods and emphasized the relevance of studying detailed precipitation effects for the arthropod diversity and ecosystem stability in arid ecosystems.

## Introduction

Climate change in terms of decreasing precipitation and altered rainfall patterns is one of the main drivers of ecosystem state shifts, as well as decreasing productivity especially in arid and semi-arid ecosystems^1,2^. Thereby, rainfall patterns can influence species composition and dynamics not only of producers, but also of organisms of higher trophic levels^3,4^. As the effect on primary production is of major concern and as predictions for sessile organisms, such as plants seem comparatively easy, there are numerous studies on the effect of climate change and altered rainfall on vegetation and plant communities (reviewed in^5^). In general, with reduced amounts of rain, perennial vegetation disappears and the amount of bare soil with ephemeral vegetation increases, disrupting the stability of ecosystems and leading to desertification^6^. Duration and amount of rain pulses affect soil moisture and therewith trophic interactions and community characteristics^7^. Thereby, rainfall pulses lead to plant recruitment and growth and increase the occurrence of organisms of higher trophic levels due to bottom-up effects^1,8^. However, most studies on the impact of precipitation regimes have mainly focused on single trophic groups, with often contradicting, taxon-specific results^9,10^, but see^11^. However, to better comprehend the effects of climate change in terms of altered rainfall on ecosystems an overarching understanding of trophic interactions is necessary^12^.

Many studies on the impacts of rainfall regimes focus on populations of larger animals, such as mammals and birds^1,13^, but see^14,15^ or species interactions between mainly mammalian herbivores or predators^4^. However, arthropods are an abundant and species-rich organism group^16^, which can make up to 90% of the animal biomass in terrestrial ecosystems^17^. Due to their small size and low dispersal ability, especially ground-dwelling arthropods respond fast and strongly to environmental changes and can therefore be used as ecological indicators^18^. Further, arthropods can contribute to a wide variety of ecosystem functions, as e.g. herbivores, predators, pollinators, seed dispersers, decomposers, and are important components of food webs and nutrient cycles^16^. In arid ecosystems, arthropods inhabit a wide range of microhabitats and are therefore important organisms stabilizing trophic interactions and ecosystem stability^19,20^. Despite of the great importance of arthropods in arid ecosystems, little is known about the effects of precipitation alterations on arthropod communities and related multi-trophic interactions^21^. Thereby rainfall influences vegetation cover, flowering and seed production and therewith herbivorous arthropods by providing food, but also microhabitat for omnivorous and predatory arthropods^9,22^. There are also direct, taxon-dependent effects of precipitation on arthropods across trophic levels^23^. For example, increasing long-term rainfall (up to 3 to 14 months before sampling) positively effects mites, ants and beetles, but negatively effects spiders, while short-term rainfall (up to 18 days) has no effect on arthropod abundance^9^. Additionally, arthropod community composition can be influenced by microhabitat characteristics, such as soil temperature, soil moisture and soil particle distribution, with arthropods being attracted by greater soil moisture, which can be related to lower coarse sand content^24^.

To disentangle the often contradicting effects of the amount and temporal distribution of rainfall on ground-dwelling arthropods, their different trophic levels and their multi-trophic interactions, we used pitfall trapping during a 6-year period in an arid savannah ecosystem. We analysed the effects of seasonal rainfall, plant cover and soil texture in terms of microhabitat availability on the activity density of herbivorous, omnivorous and predatory arthropods, detritivores and ants. Further we determined the critical window for the development of vegetation and the occurrence of the different arthropod groups in relation to rainfall. Hereby we hypothesise that:

1. Precipitation, plant cover and soil texture influence arthropod community composition.
2. Amount and timing of rainfall affect arthropod activity density differently according to their trophic group.
3. There is a cascading effect of precipitation on plant cover and subsequently on primary and secondary arthropod consumers.

## Methods

### Study area and environmental variables

The study was carried out between 2013 and 2018 in an arid savannah ecosystem on the farm Rooiklip (S 23°24′23.29’’, E 016°03′37.35’’), which is situated 1000 m a.s.l. within Namibia’s great escarpment. The climate is hot-arid, with generally erratic rainfalls, that occur highly seasonally between October and April with a distinct maximum in February and March^25^, that defines the main growing season. However, precipitation within the rainy season can also be very variable with dry spells between rainfall events^26^. Mean annual rainfall is 120 mm^27^. The decade before our study (2003–2012) was characterized by above-average annual rainfall of 208 ± 57 mm (mean ± SE) and 118 ± 39 mm during the main growing season. However, from 2013 to 2018 annual rainfall decreased by 40% at 132 ± 23 mm, while February to March rainfall decreased by 50% at 56 ± 24 mm (Supplementary Fig. S1). Mean daily air temperature was with 27.2 ± 1.2 °C in February and 27.0 ± 0.8 °C in March relatively constant over the study period (Station ID: 103—Rooisand; SASSCAL WeatherNet 2020, www.sasscalweathernet.org.). The vegetation is dominated by tussocks of perennial grasses, whose cover ranges between 1 and 50% depending on the respective precipitation^28^. This arid savannah ecosystem is sparsely interspersed with trees and small shrubs^28^ and becomes supplemented by annual grasses and herbs during the main growing season after sufficient rainfall. The soil is nutrient-poor, consists of the degradation products of the underlying schist, and has a high percentage of sand and gravel without an organic layer.

For our study, we randomly selected 30 plots of 2 m × 2 m with a distance of 111 ± 4 m (mean ± SE) between each other covering a total area of 1.1 ha. As soil surface texture can strongly influence soil moisture and therewith arthropod community composition, we used sieve analysis up to a depth of 4 cm for an area of 40 cm × 40 cm^24^ once at each plot before we studied vegetation density and arthropod activity density. In particular, we measured the amount of sand (< 0.5 cm), gravel (0.5– < 5.0 cm), cobble (5– < 15 cm), boulder (15– < 60 cm), and large boulder (≥ 60 cm) and extrapolated each gradation per plot.

Rainfall was measured on a daily base with 0.5 mm accuracy using a standard rain gauge close to the study sites (min. = 360 m, max. = 600 m apart). From the precipitation data, we calculated seasonal rainfall as the cumulative rainfall for the main growing season between February and March.

Vegetation density was characterized twice each year between March and beginning of April simultaneously to arthropod sampling by estimating the total plant cover and cover of herbs and grasses (in %), as well as counting the total number of plant species per plot. The maximum values of the respective vegetation parameter per plot per year was then used for further analysis to remove intra-seasonal variability.

### Arthropod sampling

Arthropods were sampled twice per year between March and beginning of April for one week, respectively^11^. The minimum interval between both sampling rounds was 14 days. During this time of the year, after the dry period, vegetation development is at its peak and therefore affects the development of herbivorous arthropods, but also of arthropods of all other trophic levels^26^. Arthropods were sampled using pitfall traps with a volume of 650 ml and a diameter of 90 mm, filled with 250 ml of a 50:50% ethylene glycol–water mixture and a drop detergent to reduce surface tension. Traps were buried flush with the soil surface in the centre of each plot. To prevent overflowing of traps during rainfall events plastic roofs were used. After each sampling round, arthropods were transferred into 70% ethanol. The different taxa were identified to order or family level using standardized protocols and classified according to their trophic level into herbivores, omnivores, predators and detritivores (following^29,30^, http://biodiversity.org.na/taxondisplay.php?nr=8, downloaded: 04.07.2017). Even though this very broad classification may limit informative value about community composition, a more detailed determination is often not possible, as many arthropod species have not been described taxonomically^18^. However, the use of those taxonomic levels has been successfully applied studying effects of environmental changes on arthropod communities in arid and semi-arid areas^31,32^. In case a taxon consisted of species with either herbivorous, omnivorous and/ or predatory feeding preference, such as beetles or true bugs the group was assigned to omnivores^33^. Ants were considered separately due to their behaviour as predators, scavengers, and indirect herbivores^18^. For calculations, only predominantly ground-dwelling arthropods were analysed (excluding wasps, bees, flies, midges, butterflies and moth). Larvae which were not further determinable and taxa with an overall activity density < 50 individuals over the whole 6-year study period were excluded from analysis. Activity densities of remaining taxa were calculated as the sum of activity densities per plot and year to remove intra-seasonal variability. All research activities, including arthropod sampling, were approved by the Ministry of Environment and Tourism Namibia (Permit number 1783/2013).

### Statistics

To test for multicollinearity between variables characterizing precipitation, vegetation and soil texture we performed correlation analysis (Spearman's rank correlation according to non-normality of most variables; Supplementary Table S1 online). From variable pairs with a within-group correlation coefficient of |r_s_|> 0.7^34^ only the environmental variable was used which covered a longer gradient length and which showed a better data distribution (amount of sand, boulder, large boulder, and number plant species, cover of herbs and grasses were excluded). For all analyses, R version 3.5.3^35^ was used.

To get an overview of the effects of precipitation (seasonal rainfall), plant cover and soil texture (amount of gravel and cobble) in terms of microhabitat availability on arthropod community composition, we first applied non-metric multidimensional scaling (NMDS) using the R package *vegan*^36^. From arthropod activity densities a site-taxa-matrix was calculated using Bray–Curtis dissimilarity with two dimensions. Due to varying magnitude of the occurrence of the different taxa, activity densities were normalized by the means of each column (taxa in a normal data set) making margin sum of squares equal to one^37^. Then environmental variables were fitted onto the ordination and the respective significance was assessed using 1000 permutations of variables. Stress values, squared correlation coefficient (*r*^*2*^) and *P*-values are given. Additionally, we performed a permutational multivariate analysis of variance (PERMANOVA) using the *adonis* function (R package *vegan*^36^) with the same data and explanatory variables used for NMDS to analyse to what extent these factors explain the variance of the respective arthropod communities^38,39^.

To investigate the critical window of the development of vegetation and the activity density of the different taxa and trophic levels after rainfalls we used a simple moving window approach^40^, summing up the rainfall of 7 days, 14 days and 30 days from 0 to 30 days before the start of the pitfall trapping. To obtain the highest possible resolution, each sampling round per plot and year was considered separately for this analysis. We applied generalized linear mixed-effect models (GLMM^41^) using negative binomial family (*glmer*, R package *lme4*^42^) to model the activity density of each trophic level and its determinant taxa in dependence of the respective rainfall captured by each window position. To account for the temporal structure and autocorrelation (repeated sampling of the same plot every year) we included year as fixed covariate and the factor study plot (n = 30) as a random effect^43^. For each resulting model, an adjusted *R*^*2*^-value explaining the proportion of variation by the fixed-effects factors was calculated using the R package *rsq*^44^. The resulting *R*^*2*^-values for each trophic level or taxa were plotted against the respective window position in days. A high *R*^*2*^-value, tantamount to a good correlation between the rainfall captured by the window and arthropod activity density was used as an indicator for the time frame where precipitation explained arthropod activity density best.

Lastly, the effects of seasonal rainfall and soil texture, as well as bottom-up effects along the trophic cascade on plant cover and the activity densities of herbivores, omnivores, predators, detritivores, and ants were investigated with GLMMs using the negative binomial family. Model structure and random effects were adopted from the moving window approach. According to a classical bottom-up approach, models for herbivores and detritivores contained plant cover. Models for omnivores contained plant cover and the activity density of herbivores and detritivores. Models for predators contained the activity density of herbivores, omnivores and detritivores, and models for ants contained plant cover and the activity density of herbivores and detritivores^45^. All models were checked for their goodness-of-fit. Parameter estimates, standard errors, z- and *P*-values for all GLMMs were derived from the summary table of the models. In the text and figures means and standard errors are given.

## Results

Seasonal rainfall was highly variable and ranged from 4–162 mm between February and March per year. Plant cover ranged from 0.1 to 50% per plot and was highest in the years with highest seasonal rainfall (Table 1). Soil texture consisted mainly of sand, followed by gravel, cobble, boulder, and large boulder (Supplementary Table S2 online).

**Table 1:** Annual and seasonal rainfall (in mm) for our study period, as well as distribution of plant cover and the activity density of arthropods of the different trophic levels.

Year	Seasonal rainfall (Feb.–Mar.)	*N*		Total plant cover (%)	Herbivores	Omnivores	Predators	Detritivores	Ants
2013	30.5	28	Mean ± SE	0.90 ± 0.08	6.21 ± 1.26	13.25 ± 1.81	8.79 ± 0.94	5.00 ± 1.61	147.79 ± 10.98
			Minimum	0.12	0	2	1	0	46
			Maximum	2.01	24	39	20	38	276
2014	161.5	29	Mean ± SE	24.40 ± 1.41	2.03 ± 0.32	54.55 ± 3.96	11.66 ± 0.76	34.14 ± 5.40	116.28 ± 17.06
			Minimum	13.01	0	28	3	1	23
			Maximum	45.01	8	113	19	119	375
2015	4.0	20	Mean ± SE	2.34 ± 0.34	65.90 ± 11.31	30.85 ± 5.36	10.65 ± 1.14	0.70 ± 0.23	263.30 ± 31.05
			Minimum	1.01	13	5	3	0	54
			Maximum	6.62	201	98	25	4	641
2016	48.0	25	Mean ± SE	17.70 ± 1.53	34.04 ± 3.67	38.92 ± 2.64	9.04 ± 0.90	3.64 ± 0.81	86.16 ± 13.22
			Minimum	7.11	13	19	0	0	15
			Maximum	34.20	99	69	16	19	341
2017	85.8	27	Mean ± SE	18.23 ± 1.63	81.70 ± 4.68	71.48 ± 6.25	18.48 ± 2.33	76.48 ± 10.01	49.59 ± 5.65
			Minimum	4.12	38	17	3	13	7
			Maximum	50.01	139	142	49	200	134
2018	6.0	28	Mean ± SE	0.92 ± 0.07	9.14 ± 0.92	5.11 ± 0.56	5.07 ± 0.63	8.25 ± 3.02	32.43 ± 2.98
			Minimum	0.20	3	1	0	0	15
			Maximum	2.00	25	12	13	85	82
Total	56.0 ± 24.5	157	Mean ± SE	11.08 ± 0.90	30.98 ± 3.02	5.11 ± 0.56	10.60 ± 0.60	22.49 ± 2.99	109.41 ± 8.13
			Minimum	0.12	0	1	0	0	7
			Maximum	50.01	201	142	49	200	641

Values for plant cover and arthropod activity densities were calculated for the different study plots and years.

In total, we collected 36,227 predominantly ground-dwelling arthropod individuals. Ants (Formicidae) were the taxa with the highest activity density with 19,139 individuals. We sampled 5536 herbivores including: cicadas (Cicadina), short-horned grasshoppers (Caelifera), and aphids and scale insects (Sternorrhyncha), 6141 omnivores including: beetles (Coleoptera), cockroaches (Blattodea), true bugs (Heteroptera), long-horned grasshoppers (Ensifera), and psocids or booklice (Psocoptera), 1790 predators including: spiders (Araneae), ticks and mites (Acarina), centipedes (Chilopoda), scorpions (Scorpiones), and false scorpions (Pseudoscorpiones) and 3621 detritivores comprising springtails (Collembola). The activity density of arthropods varied from year to year, and depended on the trophic level (Table 1, Supplementary Table S3 online).

**Figure 1:**
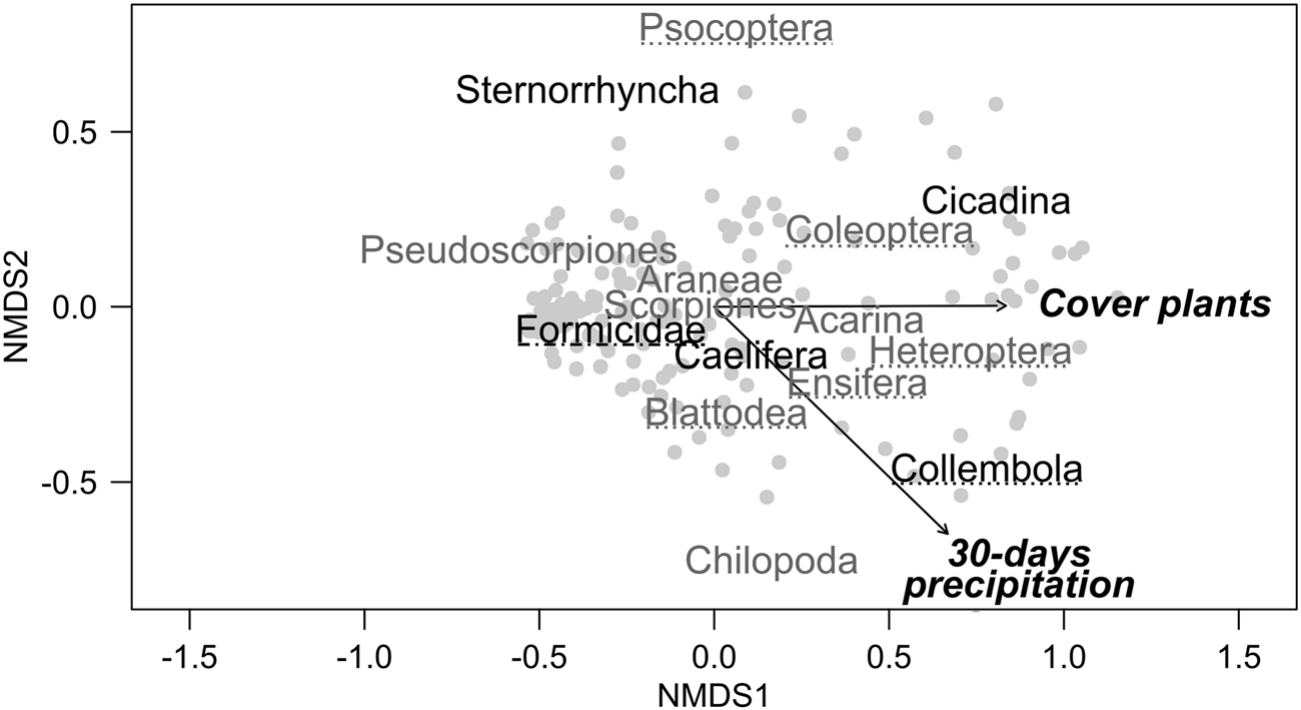
Graphical interpretation of arthropod community composition (black: herbivores; grey, dotted line: omnivores; grey: predators; black, dotted line: detritivores—springtails; black dot-dashed line: ants) by plotting site scores (light grey points) with NMDS. Only the displayed environmental variables (black arrows) influenced community composition significantly (*P* < 0.05).

NMDS fit (stress = 0.12, two convergent solutions found after 20 tries) revealed that seasonal rainfall (*r*^2^ = 0.23, *P* < 0.001) and plant cover (*r*^2^ = 0.23, *P* < 0.001) explained arthropod community composition significantly. Soil texture (gravel: *r*^2^ = 0.01, *P* = 0.45, cobble: *r*^2^ = 0.00, *P* = 0.99) had no impact on arthropod community composition. Seasonal rainfall mainly determined centipedes and detritivores in terms of springtails. Plant cover determined herbivores cicadas, as well as beetles and true bugs, which include herbivorous or predatory species. Predators including spiders, scorpions, ticks and mites, and false scorpions were not influenced by any of our measured environmental variable (Fig. 1). The PERMANOVA confirmed the influence of seasonal rainfall and plant cover on arthropod community composition, whereas the influence of soil texture components was not significant. However, rainfall and plant cover together only explain 15% of the observed differences while almost 22% could be explained by inter-annual variations (Supplementary Table S4 online).

**Figure 2:**
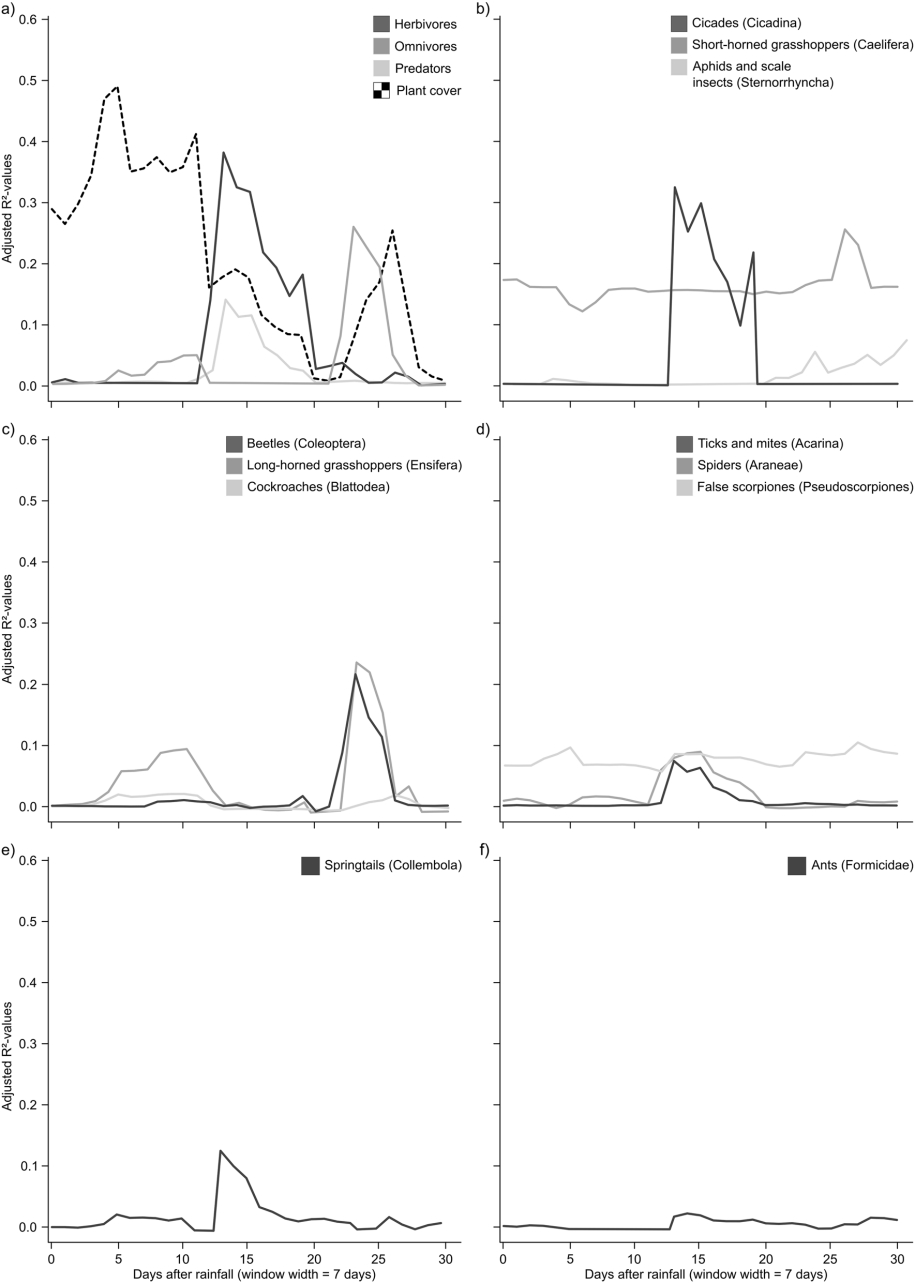
Result of the moving window approach showing adjusted *R*^2^-values to explain the proportion of variation by the fixed-effects factors from generalized linear mixed effects models. We studied the effects of the period of rainfall before pitfall trapping summed up over 7 days on (**a**) the different trophic levels of ground-dwelling arthropods, as well as plant cover, and the determinative groups that contributed most to the resulting *R*^2^-values for (**b**) herbivorous arthropods, (**c**) omnivorous arthropods, and (**d**) predatory arthropods, as well as (**e**) springtails and (**f**) ants.

In general, rainfall captured by a 7-days-width window explained plant cover and arthropod activity density best (Supplementary Table S5, S6 online), followed by the 14-days-width window (Supplementary Table S7, S8 online), while the 30-days window had practically no explanatory power (Supplementary Table S9, S10 online). The period where rainfall determined plant cover best was between 0 and 16 days, with a second peak between 24 and 27 days after the rainfall. For both herbivores and predators, highest *R*^*2*^-values were found between day 12 and day 19 after the rainfall, whereas the *R*^*2*^-values for omnivores were highest 23 to 25 days after the rainfalls (Fig. 2a). The determinative group that contributed most to the resulting *R*^*2*^-values were cicadas for the herbivores (Fig. 2b), beetles and long-horned grasshoppers for the omnivores (Fig. 2c), and ticks and mites for the predators (Fig. 2d). For detritivores in terms of springtails highest *R*^*2*^-values were found for precipitation occurring 15 days before the trapping (Fig. 2e), whereas our moving window approach did not show a response of ant activity density to any of the tested rainfall events (Fig. 2f).

Generalized linear mixed effect models analysing multi-trophic interactions revealed that seasonal rainfall increased plant cover, as well as the activity density of omnivorous arthropods and detritivores, whereas herbivores and ants decreased with seasonal rainfall. Considering the soil texture, there was a negative effect of the amount of gravel on plant cover and a positive effect on the activity density of herbivorous, omnivores and detritivores, whereas there was no further effect of soil texture on any of the other tested arthropod groups. Looking at the bottom-up effects along the food chain, we could show that plant cover increased the activity density of herbivores. Further plant cover and herbivores enhanced the activity density of omnivores. The activity density of predators was enhanced by herbivores, omnivores, as well as detritivores and ants were also enhanced by herbivores (Table 2, Supplementary Table S3 online).

**Table 2:** Effects of seasonal rainfall (Feb.–Mar.; in mm), as well as soil texture (amount of gravel and cobble in %) and bottom-up effects among plants and ground-dwelling arthropods on plant cover and arthropods’ activity density.

	Plants	Herbivores	Omnivores	Predators	Detritivores	Ants
Seasonal rainfall	0.17***	-5.76***	3.61***	n.s	6.78***	-2.64**
Gravel	-0.13*	n.s	2.40*	n.s	1.98*	n.s
Cobble	n.s	n.s	n.s	n.s	n.s	n.s
Plants	NT	3.40***	4.65***	NT	n.s	n.s
Herbivores	NT	NT	9.75***	3.23**	NT	3.63***
Omnivores	NT	NT	NT	2.01*	NT	NT
Detritivores	NT	NT	n.s	3.73***	NT	n.s

Summarized results giving estimates of generalized linear mixed-effects models: ‘n.s.‘ not significant; *P<0.05, **P < 0.01, ***P < 0.001); bold: positive effect on response variable, italic: negative effect on response variable; ‘NT’ indicates variables not tested in the respective model. For detailed model results, see Supplementary Table S3.

## Discussion

Our study corroborates the important role of seasonal rainfall and its timing for arthropod communities and their multi-trophic interactions in an arid African savannah see^9^. Looking at the arthropod community composition, we could show that especially centipedes and springtails were related to seasonal rainfall. These soft-bodied soil organisms are vulnerable to evaporation and can therefore be strongly influenced by reduced rainfall, but on the other hand, they are able to avoid negative drought effects by moving into deeper soil layers (reviewed in^21^). By contrast hard-bodied arthropods such as beetles, cicadas and true bugs where more related to increasing plant cover.

In arid regions the amount and distribution of rainfall typically varies greatly, leading to strong fluctuations in plant cover and plant species composition^28^. This, as well as population fluctuations^46^ and diapause of individual arthropod species^47,48^ also result in annually changing arthropod communities, whose composition can be explained to a limited extent by the amount of precipitation. Thus, in our study rainfall and plant cover explain only 15% of the observed differences in the community, while over 20% are due to annual differences independent of rainfall. Thereby in the long run changes in the precipitation regime can lead to changes in arthropod community composition, via changes of plant production and diversity^49^ or to lower arthropod activity and reduced recruitment and therewith to fluctuating arthropod populations^9,50^. In contrast to previous studies, where soil properties influence arthropod communities^11,24^ here we found no effects on arthropod community composition and only little effect on their activity density. In general soil texture can directly influence arthropod behaviour in terms of e.g. moving or hiding in areas with higher soil moisture during times of decreased precipitation^13,21^ but also indirectly via vegetation changes^24^. Here, the very low annual rainfall of 132 mm in comparison with other studies (73–397 mm^11^; 72–690 mm^9^; 292 mm^24^; 334 mm^14^) may have stronger direct impact on vegetation and subsequent arthropod occurrence, than microhabitat conditions in terms of soil texture. In addition, the ordination analysis, together with our moving-window approach and linear models, show that multi-trophic interactions along the food chain were also more important for predators than direct influences by the environmental variables we measured.

Generally, effects of rainfall and rainfall temporal distribution can influence various kinds of (multi-trophic) species interactions, such as facilitation, herbivory, predation, but also competition or mutualism, which still can impact community response^4,32^. Thereby, the breaking of arthropod dormancy, larval development and reproductive rates, as well as adult activity of different trophic levels in arid ecosystems are dependent on rainfall, associated with soil moisture^51^ and linked to the density of vegetation^11^. Increased soil humidity and the nutrients released by the microbial mineralization processes form the basis for the development of annual vegetation^22^. As also shown by our study, precipitation is related to increased vegetation cover and can thereby enhance aboveground primary production, plant diversity, species richness and protein content and provide habitat and food for arthropods (reviewed in^21^). Our moving window approach shows, that the critical windows of rainfall for vegetation and the different groups of arthropods are clearly different and their timing reflects the trophic cascade. In our study, plant cover showed a first pronounced response 0–16 days after rainfall and a second response to rainfall more than 24 days ago, that suggest a shift in plant community compositions probably due to facilitation among plants when water availability is intermediate^4^. Arthropods responded markedly later, when the vegetation was already developed and provides food but probably also various microhabitats due to increased structural complexity (cf.^8,9^, but see^22^). The first groups of arthropods, which simultaneously responded to rainfall in our study, were herbivores, predators and detritivores, while omnivorous arthropods responded considerably later with a time lag of about three weeks after rainfall. As omnivores in our study include true omnivorous species, but also species groups with either herbivorous or predatory feeding preference, it is likely that their activity density is mainly determined by direct effects of precipitation rather than food supply. However, omnivores may feed on plant species, which occur for the first time during the second peak of vegetation development or prey on the previously occurring herbivores and predatory arthropods. This precipitation effect, which can be used to explain multi-trophic interactions between arthropods is only to a lesser extent supported by our analysis of seasonal rainfall, as well as bottom-up effects. Our models showed strong direct effects of seasonal rainfalls not only on plants, but also on herbivores, omnivores and detritivores arthropods, as well as ants (cf.^11,45^). Thereby our moving window approach reveals, that short-term rainfall pulses (up to max. 30 days prior to survey) have shaped multi-trophic interactions among arthropods more strongly and may have outweighed seasonal patterns (cf.^14^; but see^9^). Here, bottom-up effects along the food chain could be shown for primary, as well as secondary consumers. In contrast to e.g. species-rich tropical or temperate ecosystems, where top-down effects can determine trophic interactions^52,53^, in low-productivity arid ecosystems with erratic precipitation bottom-up effects are the main driver of the trophic cascade^54,55^. Therefore, our study suggests, that the co-occurrence of especially arthropods of higher trophic levels in our study system are shaped by both, the resource pulses triggered by precipitation^7^, as well direct precipitation effects^11^. Even though our results clearly confirm the importance of rainfall on the timing of arthropod occurrence, a more detailed taxonomic classification would potentially provide more accurate results in terms of feeding adaptions and specific species interactions. This is especially critical for very diverse species groups such as beetles, where a morphological approach examining the mouthparts would better shed the light on arthropod feeding behaviour^56,57^. Here we decided to assign arthropod taxa including species with different feeding behaviour (herbivores, omnivores and predators) to omnivores (cf. ^33^) due to limitations in (taxonomic) knowledge^18^ and logistic constrains.

For detritivores, our study showed positive effects of seasonal rainfall (peaking at 15 days after rainfall) on their activity density, as well as on their position within the arthropod community (springtails^58^; but see^9^). In general springtails are restricted to moist, sheltered habitats^29^. However, they show a variety of adaptions to arid environments, such as dormancy stages, which can be rapidly broken by increasing moisture^58^, which clearly explains the short-term precipitation effects 15 days after rainfall, as well as positive effects of seasonal rainfall on detritivores. As detritivores enhance and alter soil nutrient content by converting plant material into energy and resources they are crucial for food web stability^16^. Besides the often proposed group of darkling beetles (Tenebrionidae) as indicators for environmental changes in arid ecosystems^31,46,59^, springtails can serve as good indicators of climate change due to their marked response to precipitation (c.f.^18^). Such indicators which are easy to sample and quantify^18^ are especially needed, as it is predicted that in arid and semi-arid savannah ecosystems extreme climate events will become more frequently in the future^2^.

Looking at the occurrence of ants, we only could show direct effects of seasonal precipitation but no short-term precipitation effects (critical window) on their activity density. This is in line with the often contradicting results in response of ants to climate variables (no effect of precipitation on ant activity density^11^; postive effect of annual precipitation on ant activity density^14^; increasing species richness with increasing summer precipitation^60^). Maybe other climatic factors, such as even longer periods of observed rainfall prior to the sampling or the temperature regime are more important to predict ant activity density than our tested intervals^9,14^.

In conclusion, we could show that in our arid savannah ecosystem it is important to consider short-term rainfall pulses (up to max. 30 days prior to survey) to describe the activity density of ground-dwelling arthropods and which can be used to explain multi-trophic interactions. The temporal occurrence (development) of the different arthropod groups clearly reflects the bottom-up effects. Herbivores, predators and detritivores occurred immediately after the vegetation was already developed, followed by omnivorous arthropods. Both, the activity density of detritivores and their position in the arthropod community were also strongly influences by seasonal rainfall, so they can serve as good indicator for droughts and therewith climate change. Further, seasonal precipitation had also a direct effect on the activity density of herbivorous and omnivorous arthropods, ants and also on plant cover. As precipitation strongly alters between years, also due to extreme climatic events such as El Niño Southern Oscillation^1^ even longer studies on trophic interactions but also multiple climate factors, such as rainfall magnitude and frequency^14,21^, as well as studies along larger spatial precipitation gradients are necessary to detect timescale over which rainfall and droughts affect multi-trophic interactions and ecosystem functionality. Together with changes of other climatic variables and land use changes, monitoring of arthropods across trophic levels enables to detect changes in arthropod community composition, their multi-trophic interactions and therewith ecosystem stability in time.

## Supplementary Information

Supplementary Information.

## Data Availability

All data are available from the corresponding author on reasonable request.
