# Supplementary material for: Seasonal and temporal patterns of rainfall shape arthropod community composition and multi-trophic interactions in an arid environment
Source: Sci Rep. 2022 Mar 8;12:3742. doi: 10.1038/s41598-022-07716-0 (PMC8904780; doi:10.1038/s41598-022-07716-0)
Supplement: Supplementary file 1 — Supplementary Information. [file 41598_2022_7716_MOESM1_ESM.docx]

Fischer, C., Gerstmeier, R., Wagner, T.C.: Seasonal and temporal patterns of rainfall shape arthropod community composition and multi-trophic interactions in an arid environment.


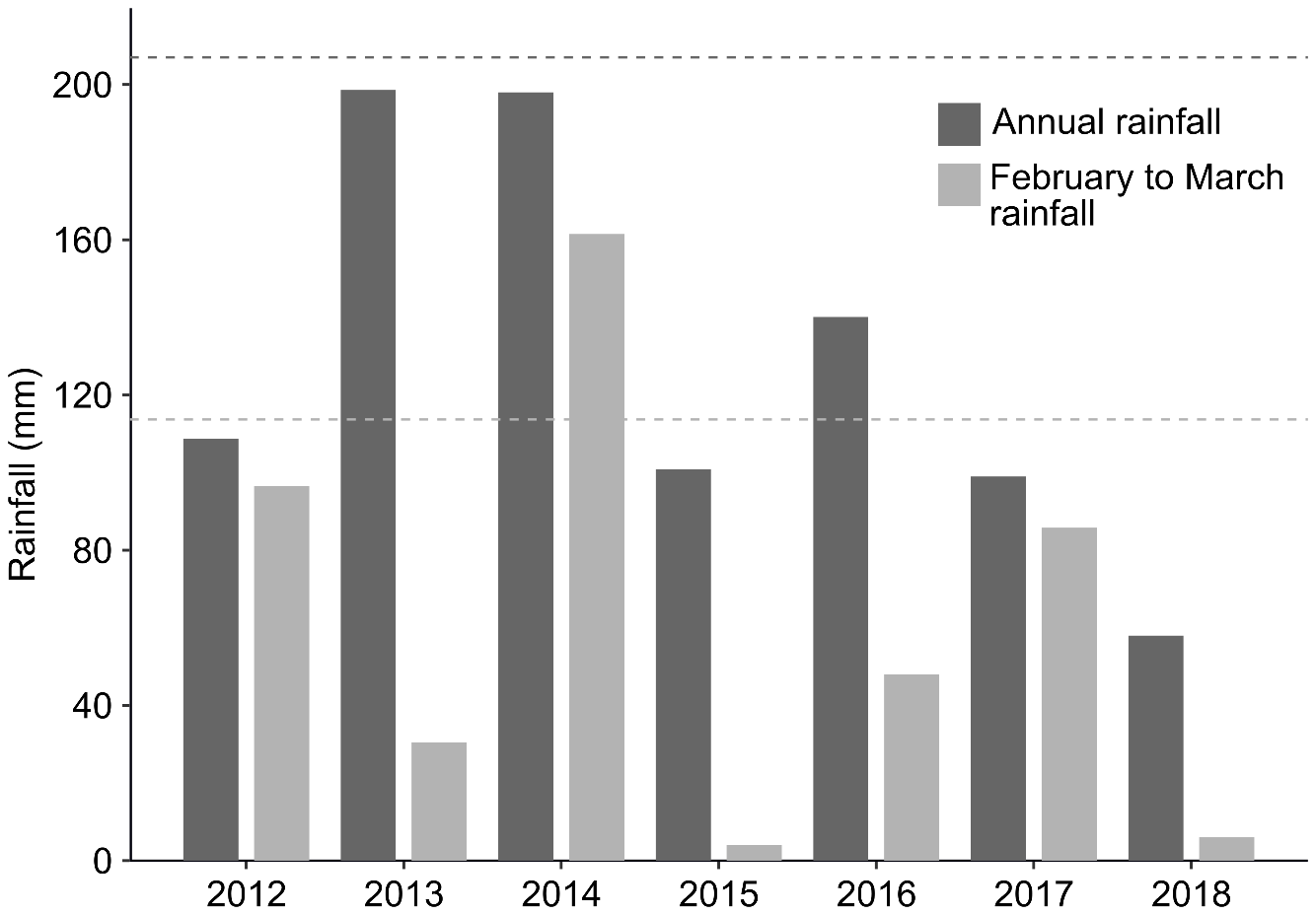


**Fig. S1**. Annual and February to March (seasonal) rainfall during the study period (and the year before the arthropod sampling started). Dark grey dashed line shows average annual rainfall and the light grey dashed line February to March rainfall the decade before the study took place.

**Table S1**. Correlation between the different biotic and abiotic environmental variables. Rainfall was calculated for the respective time frame before each sampling round. *r*- and *P*-values form Spearman’s rank correlation are given. Yellow highlighted cells (positive correlation) and bold values indicate correlation coefficient of |*r*| ≥ 0.7.

|  | **Seasonal rainfall (Feb. – Mar.)** | **Sand (%)** | **Gravel (%)** | **Cobble (%)** | **Boulder (%)** | **Large boulder (%)** | **Total plant cover (%)** | **Cover of herbs (%)** | **Cover of grasses (%)** | **No. plant species** |
| --- | --- | --- | --- | --- | --- | --- | --- | --- | --- | --- |
| **Seasonal rainfall (Feb. – Mar.)** | - |  |  |  |  |  |  |  |  |  |
| **Sand (%)** | r = -0.01  *P* = 0.90 | - |  |  |  |  |  |  |  |  |
| **Gravel (%)** | r = -0.01  *P* = 0.94 | r = 0.88  ***P* = 0.00** | - |  |  |  |  |  |  |  |
| **Cobble (%)** | r = 0.01  *P* = 0.87 | r = 0.07  *P* = 0.17 | r = -0.39  *P* = 0.00 | - |  |  |  |  |  |  |
| **Boulder (%)** | r = 0.01  *P* = 0.94 | r = -0.15  *P* = 0.01 | r = -0.15  *P* = 0.01 | r = 0.50  *P* = 0.00 |  |  |  |  |  |  |
| **Large boulder (%)** | r = 0.02  *P* = 0.82 | r = -0.53  *P* = 0.00 | r = 0.51  *P* = 0.00 | r = -0.41  *P* = 0.00 | r = -0.36  *P* = 0.00 | - |  |  |  |  |
| **Total plant cover (%)** | r = 0.76  ***P* = 0.00** | r = 0.00  *P* = 0.97 | r = -0.01  *P* = 0.83 | r = 0.03  *P* = 0.63 | r = 0.03  *P* = 0.53 | r = 0.04  *P* = 0.50 | - |  |  |  |
| **Cover of herbs (%)** | r = 0.67  ***P* = 0.00** | r = 0.06  *P* = 0.31 | r = -0.06  *P* = 0.29 | r = -0.20  *P* = 0.71 | r = -0.02  *P* = 0.69 | r = 0.06  *P* = 0.29 | r = 0.91  ***P* = 0.00** | - |  |  |
| **Cover of grasses (%)** | r = 0.80  ***P* = 0.00** | r = -0.08  *P* = 0.15 | r = 0.06  *P* = 0.26 | r = 0.04  *P* = 0.48 | r = 0.06  *P* = 0.24 | r = 0.02  *P* = 0.65 | r = 0.89  ***P* = 0.00** | r = 0.68  ***P* = 0.00** | - |  |
| **No. plant species** | r = 0.58  *P* = 0.00 | r = 0.01  *P* = 0.80 | r = -0.01  *P* = 0.82 | r = 0.01  *P* = 0.81 | r = 0.05  *P* = 0.36 | r = -0.05  *P* = 0.38 | r = 0.69  ***P* = 0.00** | r = 0.62  *P* = 0.00 | r = 0.66  ***P* = 0.00** | - |

**Table S2**. Distribution of soil parameters. Soil texture was calculated for the different study plots.

| **Parameter** | ***N*** | **Mean ± SE** | **Minimum** | **Maximum** |
| --- | --- | --- | --- | --- |
| **Sand (%)** | 30 | 42.37 ± 2.55 | 25 | 80 |
| **Gravel (%)** | 30 | 47.00 ± 2.46 | 10 | 65 |
| **Cobble (%)** | 30 | 5.48 ± 0.53 | 2 | 15 |
| **Boulder (%)** | 30 | 4.33 ± 0.52 | 1 | 15 |
| **Large boulder (%)** | 30 | 1.07 ± 0.41 | 0 | 10 |

**Table S3.** Effects of seasonal rainfall (in mm), as well as soil texture (amount of gravel and cobble in %) and bottom-up effects among plants and ground-dwelling arthropods on plant cover and arthropods’ activity density. Results of generalized linear mixed-effects models giving estimates and standard errors (SE), as well as t- or z-values and levels of significance (^*^ *P* < 0.05, ^**^ *P* < 0.01, ^***^ *P* < 0.001) for the explanatory variables from the summary table. Bold values in black indicate significant effects. NT indicates variables not tested in the respective model.

|  | Plants | | Herbivores | | Omnivores | | | Predators | | Detritivores | | Ants | |
| --- | --- | --- | --- | --- | --- | --- | --- | --- | --- | --- | --- | --- | --- |
| Parameter | Estimate ± SE | t | Estimate ± SE | z | Estimate ± SE | z | Estimate ± SE | | z | Estimate ± SE | z | Estimate ± SE | z |
| Intercept | 4.77 ± 3.13 | 1.52 | 2.55 ± 0.50 | **5.15***** | 1.97 ± 0.29 | **6.74***** | 2.40 ± 0.31 | | **7.87***** | -0.98 ± 0.66 | -1.50 | 5.86 ± 0.28 | **21.10***** |

| Seasonal rainfall (Feb. – Mar.) | 0.17 ± 0.01 | **16.82***** | -0.02 ± 0.00 | **-5.76***** | 0.01 ± 0.00 | **3.61***** | 0.00 ± 0.00 | 0.82 | 0.24 ± 0.03 | **6.78***** | -0.00 ± 0.00 | **-2.64**** | |
| --- | --- | --- | --- | --- | --- | --- | --- | --- | --- | --- | --- | --- | --- |
| Gravel | -0.13 ± 0.05 | **-2.60*** | -0.00 ± 0.01 | -0.28 | 0.01 ± 0.00 | **2.40*** | -0.01 ± 0.00 | **-**1.40 | 0.01 ± 0.01 | **1.98*** | -0.01 ± 0.00 | -1.22 | |
| Cobble | -0.26 ± 0.22 | -1.22 | 0.02 ± 0.03 | 0.58 | -0.02 ± 0.02 | -0.96 | -0.01 ± 0.02 | -0.54 | 0.01 ± 0.04 | 0.35 | -0.01 ± 0.02 | -0.65 | |
| Plants | NT | | 0.05 ± 0.01 | **3.40***** | 0.03 ± 0.01 | **4.65***** | NT | | -0.00 ± 0.01 | -0.24 | 0.01 ± 0.01 | 0.83 |  |

| Herbivores | NT | | NT | | 0.01 ± 0.00 | **9.75***** | 0.00 ± 0.00 | | **3.23**** | | NT | | | 0.01 ± 0.00 | **3.63***** |
| --- | --- | --- | --- | --- | --- | --- | --- | --- | --- | --- | --- | --- | --- | --- | --- |
| Omnivores | NT | | NT | | NT | | | 0.00 ± 0.00 | | **2.01*** | | NT | | NT | |
| Detritivores | NT | | NT | | 0.00 ± 0.00 | 0.29 | 0.00 ± 0.00 | | **3.73***** | | NT | | | 0.00 ± 0.00 | 0.92 |
| Year | 1.43 ± 0.32 | **4.53***** | 0.43 ± 0.06 | **7.65***** | -0.11 ± 0.03 | **-3.25**** | -0.08 ± 0.03 | | **-2.97**** | | 0.41 ± 0.06 | | **6.43***** | -0.41 ± 0.03 | **-12.19***** |

**Table S4.** Results of the permutational multivariate analysis of variance (PERMANOVA) for the influence of seasonal rainfall (in mm), as well as soil texture (amount of gravel and cobble in %) and plant cover on arthropod communities. Degrees of freedom (DF), sums of squares and mean sums of squares, as well as F-and *R²*-values and levels of significance (^*^ *P* < 0.05, ^**^ *P* < 0.01, ^***^ *P* < 0.001) for the explanatory variables from the summary table are given. Bold values in black indicate significant effects.

|  | DF | Sums of squares | Mean sums of squares | F | *R^2^* | *P* |
| --- | --- | --- | --- | --- | --- | --- |
| Seasonal rainfall (Feb-Mar) | 1 | 1.03 | 1.03 | 9.72 | 0.04 | **< 0.001***** |
| Gravel | 1 | 0.28 | 0.28 | 2.65 | 0.01 | **0.033*** |
| Cobble | 1 | 0.08 | 0.08 | 0.74 | 0.00 | 0.558 |
| Plant cover | 1 | 2.67 | 2.58 | 25.17 | 0.10 | **< 0.001***** |
| Year | 1 | 5.58 | 5.58 | 52.59 | 0.22 | **< 0.001***** |
| Residuals | 151 | 16.03 | 0.11 |  | 0.62 |  |
| Total | 156 | 25.68 |  |  | 1.00 |  |

**Table S5**. Raw coefficients from generalized linear mixed effects model to fit the activity density of the different ground-dwelling arthropod taxa/ plant cover in relation to the days before rainfall summed up for 7 days before the start of the pitfall trapping/ vegetation assessment.

|  | Herbivores | | | | | Omnivores | | | | | | Predators | | | | | | Detri-tivores | Ants (For-mici-dae) | Plant cover |
| --- | --- | --- | --- | --- | --- | --- | --- | --- | --- | --- | --- | --- | --- | --- | --- | --- | --- | --- | --- | --- |
| Day | Herbi-vores | Cica-das (Cica-dina) | Short-horned grass-hopper (Caeli-fera). | Aphids and scale insects (Stern-orrhyncha) | Omni-vores | | Beetle(Cole-optera) | Cock-roach-es (Blattodea) | True bugs (Heter-optera) | Long-horned grass-hopper(Ensifera) | Pso-cids or book-lice (Pso-cop-tera) | Preda-tors | Spider (Ara-neae) | Ticks and mites (Aca-rina) | Centi-pedes (Chilo-poda) | Scor-pions (Scor-piones) | False scor-pions (Pseudo-scor-piones) | Spring-tails (Coll-embo-la) |  |  |
| 0 | -0.01 | 0.00 | 0.01 | -0.05 | 0.01 | | 0.00 | 0.01 | 0.05 | 0.01 | -0.02 | 0.00 | -0.01 | 0.00 | 0.05 | 0.00 | -0.01 | 0.03 | 0.00 | 0.29 |
| 1 | -0.01 | 0.00 | 0.01 | -0.07 | 0.01 | | 0.00 | 0.01 | 0.04 | 0.01 | -0.02 | 0.00 | -0.01 | 0.00 | 0.05 | 0.00 | 0.00 | 0.03 | 0.00 | 0.27 |
| 2 | -0.01 | 0.00 | 0.01 | -0.06 | 0.01 | | 0.00 | 0.01 | 0.05 | 0.01 | -0.02 | 0.00 | -0.01 | 0.00 | 0.05 | 0.00 | -0.01 | 0.03 | 0.00 | 0.29 |
| 3 | -0.01 | 0.00 | 0.01 | -0.10 | 0.01 | | 0.00 | 0.01 | 0.05 | 0.01 | -0.02 | 0.00 | 0.00 | 0.00 | 0.05 | 0.00 | -0.01 | 0.03 | 0.00 | 0.31 |
| 4 | -0.02 | 0.00 | 0.01 | -0.12 | 0.01 | | 0.00 | 0.03 | 0.07 | 0.03 | -0.03 | 0.00 | 0.00 | -0.01 | 0.08 | 0.00 | -0.02 | 0.05 | 0.00 | 0.51 |
| 5 | -0.05 | -0.02 | -0.04 | -0.12 | 0.03 | | 0.01 | 0.05 | 0.11 | 0.05 | -0.13 | 0.01 | 0.01 | -0.01 | 0.13 | 0.03 | -0.05 | 0.08 | -0.01 | 0.75 |
| 6 | -0.04 | -0.02 | -0.06 | 0.02 | 0.03 | | 0.02 | 0.05 | 0.12 | 0.06 | NA | 0.01 | 0.01 | -0.02 | 0.13 | 0.05 | -0.03 | 0.11 | -0.02 | 0.65 |
| 7 | -0.04 | -0.02 | -0.07 | 0.01 | 0.04 | | 0.02 | 0.05 | 0.12 | 0.06 | NA | 0.01 | 0.01 | -0.02 | 0.13 | 0.05 | -0.03 | 0.11 | -0.02 | 0.66 |
| 8 | -0.03 | -0.02 | -0.06 | -0.01 | 0.04 | | 0.03 | 0.06 | 0.12 | 0.07 | NA | 0.01 | 0.01 | -0.02 | 0.13 | 0.06 | -0.04 | 0.11 | -0.02 | 0.68 |
| 9 | -0.04 | -0.02 | -0.07 | -0.06 | 0.04 | | 0.03 | 0.06 | 0.12 | 0.07 | NA | 0.01 | 0.01 | -0.03 | 0.13 | 0.06 | -0.03 | 0.10 | -0.02 | 0.65 |
| 10 | -0.04 | -0.02 | -0.08 | -0.05 | 0.05 | | 0.03 | 0.06 | 0.12 | 0.08 | NA | 0.01 | 0.01 | -0.03 | 0.15 | 0.06 | -0.03 | 0.10 | -0.02 | 0.71 |
| 11 | 0.00 | 0.00 | -0.05 | 0.11 | 0.05 | | 0.04 | 0.06 | 0.16 | 0.07 | NA | 0.02 | 0.01 | 0.01 | 0.16 | 0.04 | -0.03 | 0.16 | -0.01 | 0.84 |
| 12 | 0.06 | 0.02 | -0.03 | 0.22 | 0.07 | | 0.06 | 0.06 | 0.17 | 0.09 | NA | 0.05 | 0.04 | 0.05 | 0.01 | 0.12 | -0.03 | 0.25 | -0.06 | 0.87 |
| 13 | 0.03 | 0.03 | 0.01 | 0.09 | 0.02 | | 0.02 | 0.01 | 0.05 | 0.02 | NA | 0.03 | 0.02 | 0.05 | -0.03 | 0.02 | -0.07 | 0.06 | -0.01 | 0.34 |
| 14 | 0.03 | 0.02 | 0.00 | NA | 0.02 | | 0.01 | 0.01 | 0.05 | 0.02 | NA | 0.03 | 0.02 | 0.04 | -0.02 | 0.02 | -0.06 | 0.05 | -0.02 | 0.32 |
| 15 | 0.03 | 0.03 | 0.01 | NA | 0.02 | | 0.02 | 0.01 | 0.04 | 0.01 | 0.03 | 0.03 | 0.02 | 0.05 | -0.01 | 0.01 | -0.05 | 0.05 | -0.01 | 0.31 |
| 16 | 0.02 | 0.02 | 0.01 | NA | 0.02 | | 0.01 | 0.01 | 0.03 | 0.01 | 0.08 | 0.02 | 0.01 | 0.04 | 0.00 | 0.00 | -0.03 | 0.03 | 0.00 | 0.20 |
| 17 | 0.02 | 0.02 | 0.01 | 0.06 | 0.02 | | 0.01 | 0.01 | 0.02 | 0.01 | 0.08 | 0.02 | 0.01 | 0.04 | 0.00 | 0.00 | -0.03 | 0.03 | 0.00 | 0.17 |
| 18 | 0.02 | 0.02 | 0.01 | NA | 0.02 | | 0.01 | 0.01 | 0.02 | 0.01 | 0.09 | 0.02 | 0.01 | 0.03 | 0.00 | 0.00 | -0.03 | 0.03 | 0.00 | 0.17 |
| 19 | 0.03 | 0.03 | 0.01 | 0.05 | 0.03 | | 0.03 | 0.01 | 0.04 | 0.03 | 0.07 | 0.02 | 0.01 | 0.04 | 0.00 | 0.01 | -0.03 | 0.04 | 0.00 | 0.17 |
| 20 | 0.01 | 0.01 | 0.00 | -0.30 | 0.03 | | 0.03 | 0.02 | -0.01 | 0.03 | 0.10 | 0.00 | 0.00 | 0.00 | 0.01 | 0.00 | -0.02 | -0.04 | 0.00 | 0.04 |
| 21 | 0.01 | 0.01 | 0.00 | -0.40 | 0.03 | | 0.03 | 0.01 | 0.00 | 0.03 | 0.10 | 0.00 | 0.00 | 0.00 | 0.01 | 0.00 | -0.01 | -0.03 | 0.00 | 0.01 |
| 22 | 0.01 | 0.01 | 0.00 | -0.74 | 0.05 | | 0.05 | 0.03 | 0.01 | 0.06 | 0.12 | -0.01 | 0.00 | -0.01 | 0.04 | 0.00 | -0.02 | -0.02 | 0.00 | 0.07 |
| 23 | 0.01 | 0.01 | -0.01 | -0.69 | 0.06 | | 0.06 | 0.06 | 0.07 | 0.10 | 0.01 | -0.01 | 0.00 | -0.03 | 0.17 | 0.02 | -0.08 | 0.04 | -0.01 | 0.33 |
| 24 | 0.00 | 0.00 | -0.01 | -1.21 | 0.05 | | 0.05 | 0.05 | 0.08 | 0.08 | -0.97 | -0.01 | -0.01 | -0.03 | 0.14 | 0.02 | -0.06 | 0.06 | -0.01 | 0.41 |
| 25 | 0.00 | 0.00 | -0.01 | -0.53 | 0.04 | | 0.04 | 0.04 | 0.08 | 0.07 | -0.96 | -0.01 | -0.01 | -0.02 | 0.11 | 0.02 | -0.04 | 0.06 | -0.01 | 0.40 |
| 26 | -0.06 | -0.03 | -0.14 | -0.59 | 0.05 | | 0.03 | 0.06 | 0.12 | 0.07 | -1.07 | 0.00 | -0.01 | -0.03 | 0.13 | 0.00 | -0.04 | 0.08 | 0.00 | 0.69 |
| 27 | -0.03 | 0.01 | -0.09 | -0.52 | 0.03 | | 0.01 | 0.05 | 0.08 | 0.06 | NA | 0.00 | 0.01 | -0.02 | 0.13 | 0.02 | -0.04 | 0.05 | 0.00 | 0.37 |
| 28 | 0.00 | 0.02 | -0.05 | -0.31 | 0.01 | | 0.00 | 0.03 | 0.05 | 0.02 | -0.15 | 0.00 | 0.01 | 0.00 | 0.08 | 0.01 | -0.02 | 0.03 | 0.00 | 0.10 |
| 29 | 0.00 | 0.02 | -0.04 | -0.46 | 0.01 | | 0.00 | 0.02 | 0.03 | 0.02 | NA | 0.00 | 0.01 | 0.00 | 0.06 | 0.01 | -0.02 | 0.02 | 0.00 | 0.05 |
| 30 | 0.00 | 0.02 | -0.04 | -0.61 | 0.00 | | 0.00 | 0.01 | -0.01 | 0.01 | NA | 0.00 | 0.01 | 0.00 | 0.03 | 0.01 | -0.02 | -0.02 | 0.00 | 0.01 |

**Table S6**. R²-values from generalized linear mixed effects model to fit the activity density of the different ground-dwelling arthropod taxa/ plant cover in relation to the days before rainfall summed up for 7 days before the start of the pitfall trapping/ vegetation assessment.

|  | Herbivores | | | | | Omnivores | | | | | | Predators | | | | | | | Detriti-vores | Ants (For-mici-dae) | Plant cover |
| --- | --- | --- | --- | --- | --- | --- | --- | --- | --- | --- | --- | --- | --- | --- | --- | --- | --- | --- | --- | --- | --- |
| Day | Herbi-vores | Cica-das (Cica-dina) | Short-horned grass-hopper (Caeli-fera). | Aphids and scale insects (Stern-orrhyncha) | Omni-vores | | Beetle(Cole-optera) | Cock-roach-es (Blattodea) | True bugs (Heter-optera) | Long-horned grass-hopper(Ensifera) | Pso-cids or book-lice (Pso-cop-tera) | Preda-tors | Spider (Ara-neae) | | Ticks and mites (Aca-rina) | Centi-pedes (Chilo-poda) | Scor-pions (Scor-piones) | False scor-pions (Pseudo-scor-piones) | Spring-tails (Coll-embo-la) |  |  |
| 0 | -0.01 | -0.71 | 0.17 | 0.00 | 0.00 | | 0.01 | 0.01 | -0.20 | 0.01 | 0.01 | 0.00 | 0.01 | 0.00 | | 0.12 | 0.00 | 0.07 | 0.00 | 0.01 | 0.28 |
| 1 | 0.00 | -0.67 | 0.17 | 0.00 | 0.00 | | 0.01 | 0.01 | -0.16 | 0.01 | 0.00 | 0.00 | 0.01 | 0.00 | | 0.12 | 0.00 | 0.07 | 0.00 | 0.01 | 0.26 |
| 2 | -0.01 | -0.71 | 0.16 | 0.00 | 0.00 | | 0.01 | 0.01 | -0.26 | 0.01 | 0.00 | 0.00 | 0.01 | 0.00 | | 0.12 | 0.00 | 0.07 | 0.00 | 0.01 | 0.29 |
| 3 | -0.03 | -0.72 | 0.16 | 0.01 | 0.00 | | 0.01 | 0.01 | -0.23 | 0.02 | 0.00 | 0.00 | 0.01 | 0.00 | | 0.15 | 0.00 | 0.08 | 0.00 | 0.01 | 0.34 |
| 4 | -0.05 | -0.74 | 0.16 | 0.01 | 0.01 | | 0.01 | 0.02 | -0.06 | 0.03 | 0.01 | 0.00 | 0.00 | 0.00 | | 0.21 | 0.00 | 0.09 | 0.01 | 0.00 | 0.46 |
| 5 | -0.05 | -0.64 | 0.13 | 0.01 | 0.02 | | 0.01 | 0.03 | 0.13 | 0.06 | 0.00 | 0.00 | 0.01 | 0.00 | | 0.23 | 0.01 | 0.10 | 0.02 | -0.01 | 0.48 |
| 6 | -0.01 | -0.51 | 0.12 | 0.01 | 0.02 | | 0.01 | 0.02 | 0.06 | 0.07 | NA | 0.00 | 0.02 | 0.00 | | 0.08 | 0.02 | 0.07 | 0.02 | -0.05 | 0.35 |
| 7 | -0.01 | -0.54 | 0.14 | 0.00 | 0.02 | | 0.01 | 0.02 | 0.06 | 0.07 | NA | 0.00 | 0.02 | 0.00 | | 0.08 | 0.02 | 0.07 | 0.02 | -0.05 | 0.35 |
| 8 | -0.04 | -0.71 | 0.16 | 0.00 | 0.04 | | 0.02 | 0.03 | 0.08 | 0.09 | NA | 0.00 | 0.02 | 0.00 | | 0.08 | 0.03 | 0.07 | 0.01 | -0.05 | 0.37 |
| 9 | -0.02 | -0.60 | 0.16 | 0.00 | 0.04 | | 0.02 | 0.03 | 0.05 | 0.10 | NA | 0.00 | 0.01 | 0.00 | | 0.08 | 0.03 | 0.07 | 0.01 | -0.05 | 0.34 |
| 10 | -0.02 | -0.62 | 0.16 | 0.00 | 0.05 | | 0.02 | 0.03 | 0.08 | 0.10 | NA | 0.00 | 0.01 | 0.00 | | 0.12 | 0.02 | 0.07 | 0.01 | -0.04 | 0.35 |
| 11 | -0.01 | -0.73 | 0.15 | -0.19 | 0.05 | | 0.02 | 0.03 | 0.24 | 0.07 | NA | 0.01 | 0.01 | 0.00 | | 0.22 | 0.01 | 0.07 | -0.01 | -0.01 | 0.41 |
| 12 | 0.13 | -0.40 | 0.15 | -0.09 | 0.00 | | 0.01 | 0.01 | -0.20 | 0.03 | NA | 0.02 | 0.06 | 0.01 | | 0.00 | 0.04 | 0.06 | -0.42 | -0.04 | 0.16 |
| 13 | 0.38 | 0.32 | 0.16 | 0.00 | -0.01 | | 0.01 | 0.00 | 0.13 | 0.01 | NA | 0.14 | 0.08 | 0.08 | | -0.01 | 0.01 | 0.09 | 0.12 | 0.02 | 0.17 |
| 14 | 0.32 | 0.25 | 0.16 | NA | 0.00 | | 0.01 | 0.00 | 0.09 | 0.01 | NA | 0.11 | 0.09 | 0.06 | | -0.01 | 0.01 | 0.09 | 0.10 | 0.03 | 0.19 |
| 15 | 0.31 | 0.29 | 0.16 | NA | -0.01 | | 0.01 | 0.00 | 0.07 | 0.01 | 0.00 | 0.11 | 0.09 | 0.07 | | 0.00 | 0.01 | 0.09 | 0.08 | 0.02 | 0.17 |
| 16 | 0.21 | 0.20 | 0.15 | NA | 0.00 | | 0.01 | 0.00 | 0.03 | 0.00 | 0.03 | 0.06 | 0.06 | 0.03 | | 0.00 | 0.00 | 0.08 | 0.03 | 0.02 | 0.11 |
| 17 | 0.19 | 0.17 | 0.15 | -0.01 | 0.00 | | 0.01 | 0.00 | 0.02 | 0.00 | 0.09 | 0.05 | 0.05 | 0.03 | | 0.00 | 0.00 | 0.08 | 0.02 | 0.01 | 0.09 |
| 18 | 0.14 | 0.09 | 0.15 | NA | 0.00 | | 0.01 | 0.00 | 0.01 | 0.00 | 0.12 | 0.02 | 0.04 | 0.01 | | 0.00 | 0.00 | 0.08 | 0.01 | 0.01 | 0.08 |
| 19 | 0.18 | 0.21 | 0.15 | 0.00 | -0.03 | | 0.02 | 0.00 | -0.04 | 0.01 | 0.04 | 0.02 | 0.03 | 0.01 | | 0.00 | 0.00 | 0.08 | 0.01 | 0.02 | 0.08 |
| 20 | 0.02 | -0.33 | 0.15 | 0.01 | -0.06 | | 0.00 | 0.00 | 0.00 | -0.05 | 0.25 | 0.00 | 0.00 | 0.00 | | -0.01 | 0.00 | 0.07 | 0.01 | 0.01 | 0.01 |
| 21 | 0.03 | -0.33 | 0.15 | 0.01 | -0.03 | | 0.01 | 0.00 | 0.00 | -0.04 | 0.22 | 0.00 | 0.00 | 0.00 | | -0.01 | 0.00 | 0.07 | 0.01 | 0.01 | 0.00 |
| 22 | 0.03 | -0.31 | 0.15 | 0.02 | 0.08 | | 0.10 | 0.00 | 0.00 | 0.01 | 0.06 | 0.00 | 0.00 | 0.01 | | -0.01 | 0.00 | 0.07 | 0.01 | 0.01 | 0.01 |
| 23 | 0.01 | -0.51 | 0.16 | 0.06 | 0.26 | | 0.22 | 0.01 | -0.25 | 0.24 | 0.00 | 0.00 | 0.00 | 0.01 | | 0.04 | 0.01 | 0.09 | 0.01 | 0.01 | 0.07 |
| 24 | -0.01 | -0.64 | 0.17 | 0.02 | 0.23 | | 0.15 | 0.01 | -1.36 | 0.23 | -0.01 | 0.00 | 0.00 | 0.01 | | 0.13 | 0.01 | 0.09 | -0.04 | 0.00 | 0.14 |
| 25 | -0.03 | -0.70 | 0.17 | 0.03 | 0.19 | | 0.12 | 0.02 | -0.60 | 0.16 | 0.00 | 0.00 | 0.00 | 0.01 | | 0.18 | 0.01 | 0.09 | -0.01 | 0.00 | 0.16 |
| 26 | 0.01 | -0.51 | 0.25 | 0.04 | 0.05 | | 0.02 | 0.03 | 0.10 | 0.02 | 0.00 | 0.00 | 0.00 | 0.00 | | 0.24 | 0.00 | 0.09 | 0.02 | 0.01 | 0.25 |
| 27 | 0.01 | -0.90 | 0.23 | 0.05 | 0.02 | | 0.01 | 0.02 | -0.01 | 0.04 | NA | 0.00 | 0.01 | 0.01 | | 0.09 | 0.01 | 0.11 | 0.00 | 0.01 | 0.14 |
| 28 | -0.01 | -0.94 | 0.16 | 0.04 | 0.00 | | 0.01 | 0.01 | -0.60 | -0.01 | 0.00 | 0.00 | 0.01 | 0.00 | | -0.25 | 0.00 | 0.10 | 0.00 | 0.02 | 0.03 |
| 29 | -0.01 | -0.85 | 0.16 | 0.05 | 0.00 | | 0.01 | 0.01 | -0.14 | -0.01 | NA | 0.00 | 0.01 | 0.00 | | -0.28 | 0.00 | 0.09 | 0.00 | 0.02 | 0.01 |
| 30 | -0.01 | -0.83 | 0.16 | 0.07 | 0.00 | | 0.01 | 0.01 | 0.00 | 0.00 | NA | 0.00 | 0.01 | 0.00 | | 0.01 | 0.00 | 0.09 | 0.01 | 0.02 | 0.00 |

**Table S7**. Raw coefficients from generalized linear mixed effects model to fit the activity density of the different ground-dwelling arthropod taxa/ plant cover in relation to the days before rainfall summed up for 14 days before the start of the pitfall trapping/ vegetation assessment.

|  | Herbivores | | | | | Omnivores | | | | | | Predators | | | | | | | Detriti-vores | Ants (For-mici-dae) | Plant cover |
| --- | --- | --- | --- | --- | --- | --- | --- | --- | --- | --- | --- | --- | --- | --- | --- | --- | --- | --- | --- | --- | --- |
| Day | Herbi-vores | Cica-das (Cica-dina) | Short-horned grass-hopper (Caeli-fera). | Aphids and scale insects (Stern-orrhyncha) | Omni-vores | | Beetle(Cole-optera) | Cock-roach-es (Blattodea) | True bugs (Heter-optera) | Long-horned grass-hopper(Ensifera) | Pso-cids or book-lice (Pso-coptera) | Preda-tors | Spider (Ara-neae) | Ticks and mites (Aca-rina) | Centi-pedes (Chilo-poda) | Scor-pions (Scor-piones) | | False scor-pions (Pseudo-scor-piones) | Spring-tails (Coll-embo-la) |  |  |
| 0 | -0.01 | 0.00 | 0.00 | -0.03 | 0.01 | | 0.00 | 0.01 | 0.04 | 0.01 | -0.02 | 0.00 | 0.00 | 0.00 | 0.05 | 0.00 | -0.01 | | 0.00 | 0.03 | 0.28 |
| 1 | -0.01 | -0.01 | 0.00 | -0.05 | 0.01 | | 0.00 | 0.01 | 0.04 | 0.02 | -0.02 | 0.00 | 0.00 | -0.01 | 0.05 | 0.01 | -0.01 | | 0.00 | 0.03 | 0.28 |
| 2 | -0.01 | 0.00 | 0.00 | -0.04 | 0.01 | | 0.00 | 0.02 | 0.04 | 0.02 | -0.02 | 0.00 | 0.00 | 0.00 | 0.05 | 0.01 | -0.01 | | 0.00 | 0.03 | 0.29 |
| 3 | -0.01 | 0.00 | 0.00 | -0.07 | 0.01 | | 0.00 | 0.02 | 0.04 | 0.02 | -0.03 | 0.00 | 0.00 | 0.00 | 0.05 | 0.00 | -0.01 | | 0.00 | 0.03 | 0.29 |
| 4 | -0.01 | 0.00 | 0.00 | -0.03 | 0.01 | | 0.01 | 0.02 | 0.06 | 0.02 | -0.04 | 0.00 | 0.00 | 0.00 | 0.06 | 0.01 | -0.02 | | 0.00 | 0.05 | 0.38 |
| 5 | -0.02 | -0.01 | -0.03 | 0.04 | 0.03 | | 0.02 | 0.04 | 0.10 | 0.05 | NA | 0.01 | 0.01 | 0.00 | 0.10 | 0.04 | -0.03 | | -0.02 | 0.10 | 0.59 |
| 6 | 0.01 | 0.01 | 0.00 | 0.06 | 0.02 | | 0.01 | 0.02 | 0.06 | 0.03 | NA | 0.02 | 0.01 | 0.02 | 0.05 | 0.02 | -0.03 | | -0.01 | 0.07 | 0.33 |
| 7 | 0.01 | 0.01 | 0.00 | 0.06 | 0.02 | | 0.01 | 0.02 | 0.06 | 0.02 | NA | 0.02 | 0.01 | 0.02 | 0.04 | 0.02 | -0.03 | | -0.02 | 0.07 | 0.30 |
| 8 | 0.01 | 0.01 | 0.00 | NA | 0.02 | | 0.02 | 0.02 | 0.06 | 0.03 | 0.00 | 0.02 | 0.01 | 0.03 | 0.05 | 0.02 | -0.03 | | -0.01 | 0.07 | 0.31 |
| 9 | 0.01 | 0.01 | 0.00 | 0.05 | 0.02 | | 0.02 | 0.02 | 0.05 | 0.02 | 0.06 | 0.02 | 0.01 | 0.02 | 0.05 | 0.01 | -0.03 | | -0.01 | 0.06 | 0.26 |
| 10 | 0.01 | 0.01 | 0.00 | 0.05 | 0.02 | | 0.02 | 0.02 | 0.05 | 0.02 | 0.06 | 0.01 | 0.01 | 0.02 | 0.05 | 0.01 | -0.03 | | -0.01 | 0.06 | 0.25 |
| 11 | 0.01 | 0.01 | 0.00 | NA | 0.03 | | 0.02 | 0.02 | 0.06 | 0.02 | 0.06 | 0.02 | 0.01 | 0.03 | 0.06 | 0.01 | -0.03 | | 0.00 | 0.06 | 0.27 |
| 12 | 0.03 | 0.02 | 0.00 | 0.05 | 0.03 | | 0.03 | 0.01 | 0.04 | 0.03 | 0.07 | 0.02 | 0.01 | 0.03 | 0.00 | 0.01 | -0.02 | | -0.01 | 0.04 | 0.18 |
| 13 | 0.03 | 0.03 | 0.01 | 0.04 | 0.03 | | 0.03 | 0.01 | 0.03 | 0.02 | 0.07 | 0.02 | 0.01 | 0.03 | 0.00 | 0.01 | -0.03 | | -0.01 | 0.03 | 0.17 |
| 14 | 0.03 | 0.02 | 0.00 | 0.04 | 0.03 | | 0.03 | 0.01 | 0.03 | 0.03 | 0.07 | 0.02 | 0.01 | 0.03 | 0.00 | 0.01 | -0.03 | | -0.01 | 0.03 | 0.17 |
| 15 | 0.02 | 0.02 | 0.00 | 0.04 | 0.03 | | 0.03 | 0.01 | 0.04 | 0.03 | 0.07 | 0.02 | 0.01 | 0.03 | 0.01 | 0.01 | -0.03 | | -0.01 | 0.04 | 0.19 |
| 16 | 0.02 | 0.02 | 0.00 | 0.04 | 0.04 | | 0.03 | 0.02 | 0.06 | 0.03 | 0.06 | 0.02 | 0.01 | 0.03 | 0.03 | 0.01 | -0.04 | | -0.01 | 0.05 | 0.22 |
| 17 | 0.02 | 0.02 | 0.00 | 0.03 | 0.04 | | 0.03 | 0.02 | 0.07 | 0.04 | 0.07 | 0.02 | 0.01 | 0.03 | 0.07 | 0.01 | -0.04 | | 0.00 | 0.06 | 0.25 |
| 18 | 0.02 | 0.02 | 0.00 | 0.02 | 0.04 | | 0.03 | 0.03 | 0.07 | 0.04 | NA | 0.01 | 0.01 | 0.02 | 0.10 | 0.01 | -0.04 | | 0.00 | 0.06 | 0.27 |
| 19 | 0.02 | 0.02 | 0.00 | 0.02 | 0.04 | | 0.03 | 0.03 | 0.08 | 0.04 | NA | 0.01 | 0.01 | 0.03 | NA | 0.01 | -0.04 | | 0.00 | 0.07 | 0.27 |
| 20 | 0.00 | 0.01 | -0.01 | -0.37 | 0.03 | | 0.03 | 0.03 | 0.02 | 0.04 | NA | 0.00 | 0.01 | -0.01 | 0.12 | 0.01 | -0.03 | | 0.00 | 0.00 | 0.16 |
| 21 | 0.00 | 0.02 | -0.01 | -0.41 | 0.02 | | 0.02 | 0.02 | 0.01 | 0.03 | 0.07 | 0.00 | 0.01 | -0.01 | 0.08 | 0.00 | -0.02 | | 0.00 | -0.01 | 0.07 |
| 22 | 0.00 | 0.02 | -0.01 | -0.48 | 0.03 | | 0.02 | 0.02 | 0.02 | 0.04 | 0.04 | 0.00 | 0.01 | -0.01 | 0.08 | 0.01 | -0.02 | | 0.00 | 0.00 | 0.06 |
| 23 | 0.00 | 0.02 | -0.02 | -0.61 | 0.02 | | 0.02 | 0.03 | 0.03 | 0.04 | -0.03 | 0.00 | 0.01 | -0.01 | 0.06 | 0.01 | -0.02 | | 0.00 | 0.01 | 0.07 |
| 24 | 0.00 | 0.01 | -0.02 | -0.47 | 0.02 | | 0.02 | 0.02 | 0.03 | 0.04 | NA | 0.00 | 0.01 | -0.01 | 0.06 | 0.01 | -0.02 | | 0.00 | 0.02 | 0.10 |
| 25 | 0.00 | 0.01 | -0.02 | -0.31 | 0.02 | | 0.02 | 0.02 | 0.04 | 0.03 | NA | 0.00 | 0.00 | -0.01 | 0.06 | 0.01 | -0.02 | | 0.00 | 0.02 | 0.13 |
| 26 | 0.00 | 0.01 | -0.04 | -0.50 | 0.01 | | 0.01 | 0.02 | 0.04 | 0.02 | NA | 0.00 | 0.00 | 0.00 | 0.05 | 0.00 | -0.02 | | 0.00 | 0.02 | 0.12 |
| 27 | 0.00 | 0.01 | -0.04 | -0.53 | 0.01 | | 0.01 | 0.02 | 0.04 | 0.02 | NA | 0.00 | 0.00 | 0.00 | 0.05 | 0.00 | -0.02 | | 0.00 | 0.02 | 0.12 |
| 28 | 0.00 | 0.01 | -0.03 | -0.49 | 0.01 | | 0.00 | 0.02 | 0.04 | 0.02 | NA | 0.00 | 0.00 | 0.00 | 0.05 | 0.00 | -0.02 | | 0.00 | 0.02 | 0.12 |
| 29 | 0.00 | 0.01 | -0.03 | -0.13 | 0.01 | | 0.00 | 0.02 | 0.04 | 0.02 | NA | 0.00 | 0.00 | 0.00 | 0.05 | 0.00 | -0.02 | | 0.00 | 0.03 | 0.12 |
| 30 | 0.00 | 0.01 | -0.03 | -0.13 | 0.01 | | 0.00 | 0.02 | 0.05 | 0.02 | NA | 0.00 | 0.00 | 0.00 | 0.05 | 0.00 | -0.02 | | 0.00 | 0.03 | 0.11 |

**Table S8**. R²-values from generalized linear mixed effects model to fit the activity density of the different ground-dwelling arthropod taxa/ plant cover in relation to the days before rainfall summed up for 14 days before the start of the pitfall trapping/ vegetation assessment.

|  | Herbivores | | | | | Omnivores | | | | | | Predators | | | | | | Detriti-vores | Ants (For-mici-dae) | Plant cover |
| --- | --- | --- | --- | --- | --- | --- | --- | --- | --- | --- | --- | --- | --- | --- | --- | --- | --- | --- | --- | --- |
| Day | Herbi-vores | Cica-das (Cica-dina) | Short-horned grass-hopper (Caeli-fera). | Aphids and scale insects (Stern-orrhyncha) | Omni-vores | | Beetle(Cole-optera) | Cock-roach-es (Blattodea) | True bugs (Heter-optera) | Long-horned grass-hopper(Ensifera) | Pso-cids or book-lice (Pso-cop-tera) | Preda-tors | Spider (Ara-neae) | Ticks and mites (Aca-rina) | Centi-pedes (Chilo-poda) | Scor-pions (Scor-piones) | False scor-pions (Pseudo-scor-piones) | Spring-tails (Coll-embo-la) |  |  |
| 0 | -0.01 | -0.69 | 0.16 | 0.00 | 0.01 | | 0.01 | 0.02 | 0.06 | 0.03 | -0.07 | 0.00 | 0.00 | 0.00 | 0.21 | 0.00 | 0.07 | 0.01 | 0.00 | 0.43 |
| 1 | -0.01 | -0.66 | 0.16 | 0.00 | 0.01 | | 0.01 | 0.02 | 0.04 | 0.04 | -0.07 | 0.00 | 0.00 | 0.00 | 0.21 | 0.01 | 0.07 | 0.01 | -0.01 | 0.42 |
| 2 | -0.02 | -0.71 | 0.16 | 0.00 | 0.02 | | 0.01 | 0.02 | 0.05 | 0.04 | -0.09 | 0.00 | 0.00 | 0.00 | 0.21 | 0.00 | 0.07 | 0.01 | 0.00 | 0.44 |
| 3 | -0.03 | -0.72 | 0.16 | 0.01 | 0.02 | | 0.01 | 0.02 | 0.06 | 0.05 | 0.01 | 0.00 | 0.00 | 0.00 | 0.22 | 0.00 | 0.08 | 0.01 | 0.00 | 0.46 |
| 4 | -0.06 | -0.75 | 0.15 | 0.00 | 0.03 | | 0.01 | 0.02 | 0.17 | 0.05 | -0.06 | 0.00 | 0.00 | 0.00 | 0.23 | 0.01 | 0.08 | 0.03 | 0.00 | 0.54 |
| 5 | -0.08 | -0.80 | 0.14 | 0.00 | 0.03 | | 0.01 | 0.03 | 0.14 | 0.08 | NA | 0.01 | 0.02 | 0.00 | 0.09 | 0.02 | 0.08 | 0.03 | -0.04 | 0.48 |
| 6 | 0.16 | -0.13 | 0.15 | -0.42 | -0.01 | | 0.01 | 0.01 | -0.02 | 0.03 | NA | 0.06 | 0.07 | 0.03 | 0.01 | 0.01 | 0.08 | 0.10 | 0.00 | 0.35 |
| 7 | 0.13 | -0.19 | 0.15 | -1.12 | -0.01 | | 0.01 | 0.01 | -0.40 | 0.03 | NA | 0.05 | 0.08 | 0.02 | 0.00 | 0.02 | 0.08 | -0.25 | -0.01 | 0.34 |
| 8 | 0.16 | -0.02 | 0.16 | NA | -0.06 | | 0.00 | 0.02 | -0.87 | 0.03 | 0.00 | 0.05 | 0.08 | 0.02 | -0.03 | 0.02 | 0.08 | -0.05 | 0.00 | 0.34 |
| 9 | 0.13 | 0.01 | 0.16 | -0.09 | -0.03 | | 0.01 | 0.01 | -0.19 | 0.03 | 0.01 | 0.04 | 0.07 | 0.01 | -0.03 | 0.01 | 0.08 | 0.04 | 0.01 | 0.28 |
| 10 | 0.13 | 0.02 | 0.16 | -0.04 | -0.01 | | 0.01 | 0.01 | -0.07 | 0.02 | 0.02 | 0.04 | 0.06 | 0.01 | -0.01 | 0.01 | 0.08 | 0.04 | 0.01 | 0.26 |
| 11 | 0.14 | 0.04 | 0.16 | NA | -0.01 | | 0.01 | 0.01 | -0.07 | 0.02 | 0.02 | 0.04 | 0.05 | 0.02 | 0.00 | 0.00 | 0.08 | 0.03 | 0.01 | 0.25 |
| 12 | 0.22 | 0.23 | 0.15 | 0.00 | 0.00 | | 0.03 | 0.01 | 0.00 | 0.03 | 0.03 | 0.03 | 0.04 | 0.02 | 0.00 | 0.01 | 0.07 | 0.02 | 0.02 | 0.12 |
| 13 | 0.21 | 0.24 | 0.15 | 0.00 | 0.00 | | 0.03 | 0.01 | 0.00 | 0.03 | 0.03 | 0.03 | 0.05 | 0.02 | 0.00 | 0.01 | 0.08 | 0.02 | 0.02 | 0.11 |
| 14 | 0.20 | 0.23 | 0.15 | 0.00 | 0.02 | | 0.05 | 0.01 | 0.00 | 0.04 | 0.03 | 0.03 | 0.04 | 0.02 | 0.00 | 0.01 | 0.08 | 0.02 | 0.02 | 0.10 |
| 15 | 0.20 | 0.22 | 0.15 | 0.00 | 0.00 | | 0.03 | 0.01 | -0.01 | 0.03 | 0.03 | 0.03 | 0.04 | 0.02 | 0.00 | 0.01 | 0.09 | 0.02 | 0.02 | 0.12 |
| 16 | 0.18 | 0.21 | 0.15 | 0.00 | 0.01 | | 0.04 | 0.01 | -0.08 | 0.04 | 0.02 | 0.03 | 0.04 | 0.02 | 0.00 | 0.01 | 0.10 | 0.02 | 0.02 | 0.17 |
| 17 | 0.17 | 0.20 | 0.15 | 0.00 | 0.02 | | 0.04 | 0.01 | -0.13 | 0.04 | 0.02 | 0.03 | 0.04 | 0.02 | 0.01 | 0.01 | 0.10 | 0.02 | 0.01 | 0.22 |
| 18 | 0.12 | 0.12 | 0.15 | 0.00 | 0.04 | | 0.05 | 0.01 | -0.41 | 0.05 | NA | 0.02 | 0.03 | 0.01 | 0.02 | 0.01 | 0.11 | -0.04 | 0.01 | 0.24 |
| 19 | 0.14 | 0.15 | 0.15 | 0.00 | 0.05 | | 0.05 | 0.01 | -0.76 | 0.04 | NA | 0.02 | 0.02 | 0.01 | NA | 0.00 | 0.10 | -0.13 | 0.01 | 0.22 |
| 20 | -0.02 | -0.36 | 0.17 | 0.06 | 0.04 | | 0.04 | 0.01 | -0.01 | -0.02 | NA | 0.00 | 0.01 | 0.01 | 0.02 | 0.00 | 0.10 | 0.01 | 0.01 | 0.08 |
| 21 | 0.00 | -0.30 | 0.17 | 0.08 | 0.00 | | 0.03 | 0.01 | 0.00 | -0.05 | 0.04 | 0.00 | 0.01 | 0.01 | 0.01 | 0.00 | 0.09 | 0.01 | 0.02 | 0.02 |
| 22 | 0.00 | -0.31 | 0.18 | 0.08 | -0.11 | | 0.01 | 0.01 | 0.00 | -0.40 | 0.00 | 0.00 | 0.01 | 0.01 | -0.17 | 0.01 | 0.10 | 0.01 | 0.02 | 0.02 |
| 23 | -0.01 | -0.46 | 0.19 | 0.20 | -0.04 | | 0.03 | 0.01 | -0.01 | -0.78 | 0.00 | 0.00 | 0.01 | 0.00 | -0.03 | 0.01 | 0.10 | 0.01 | 0.01 | 0.02 |
| 24 | -0.01 | -0.51 | 0.19 | 0.06 | 0.01 | | 0.03 | 0.01 | -0.05 | -0.34 | NA | 0.00 | 0.01 | 0.00 | 0.00 | 0.01 | 0.10 | 0.00 | 0.01 | 0.04 |
| 25 | -0.02 | -0.54 | 0.19 | 0.04 | 0.04 | | 0.03 | 0.01 | -0.04 | -0.06 | NA | 0.00 | 0.00 | 0.00 | 0.07 | 0.01 | 0.10 | 0.01 | 0.02 | 0.08 |
| 26 | -0.01 | -0.86 | 0.20 | 0.07 | 0.01 | | 0.01 | 0.01 | -0.24 | -0.01 | NA | 0.00 | 0.00 | 0.00 | 0.07 | 0.00 | 0.10 | 0.00 | 0.02 | 0.07 |
| 27 | -0.01 | -0.84 | 0.19 | 0.08 | 0.01 | | 0.01 | 0.01 | -0.28 | 0.00 | NA | 0.00 | 0.00 | 0.00 | 0.07 | 0.00 | 0.10 | 0.00 | 0.03 | 0.07 |
| 28 | -0.01 | -0.85 | 0.16 | 0.08 | 0.00 | | 0.01 | 0.01 | -0.38 | 0.00 | NA | 0.00 | 0.00 | 0.00 | 0.07 | 0.00 | 0.10 | 0.00 | 0.03 | 0.07 |
| 29 | -0.01 | -0.75 | 0.16 | 0.02 | 0.00 | | 0.01 | 0.01 | -1.06 | 0.00 | NA | 0.00 | 0.00 | 0.00 | 0.05 | 0.00 | 0.10 | -0.01 | 0.03 | 0.07 |
| 30 | -0.01 | -0.73 | 0.16 | 0.02 | 0.00 | | 0.01 | 0.01 | -3.49 | -0.01 | NA | 0.00 | 0.00 | 0.00 | 0.00 | 0.00 | 0.09 | -0.03 | 0.02 | 0.05 |

**Table S9**. Raw coefficients from generalized linear mixed effects model to fit the activity density of the different ground-dwelling arthropod taxa/ plant cover in relation to the days before rainfall summed up for 30 days before the start of the pitfall trapping/ vegetation assessment.

|  | Herbivores | | | | | Omnivores | | | | | | | Predators | | | | | | Detriti-vores | Ants (For-mici-dae) | Plant cover |
| --- | --- | --- | --- | --- | --- | --- | --- | --- | --- | --- | --- | --- | --- | --- | --- | --- | --- | --- | --- | --- | --- |
| Day | Herbi-vores | Cica-das (Cica-dina) | Short-horned grass-hopper (Caeli-fera). | Aphids and scale insects (Stern-orrhyncha) | Omni-vores | | Beetle(Cole-optera) | Cock-roach-es (Blattodea) | True bugs (Heter-optera) | Long-horned grass-hopper(Ensifera) | Pso-cids or book-lice (Pso-cop-tera) | Preda-tors | | Spider (Ara-neae) | Ticks and mites (Aca-rina) | Centi-pedes (Chilo-poda) | Scor-pions (Scor-piones) | False scor-pions (Pseudo-scor-piones) | Spring-tails (Coll-embo-la) |  |  |
| 0 | 0.01 | 0.01 | 0.00 | 0.01 | 0.02 | | 0.01 | 0.02 | 0.06 | 0.02 | 0.03 | 0.01 | | 0.00 | 0.01 | 0.05 | 0.01 | -0.01 | 0.04 | 0.00 | 0.28 |
| 1 | 0.00 | 0.01 | 0.00 | 0.00 | 0.02 | | 0.01 | 0.02 | 0.06 | 0.02 | 0.03 | 0.01 | | 0.00 | 0.01 | 0.05 | 0.01 | -0.01 | 0.04 | 0.00 | 0.27 |
| 2 | 0.00 | 0.01 | 0.00 | 0.00 | 0.02 | | 0.01 | 0.02 | 0.06 | 0.02 | 0.02 | 0.01 | | 0.00 | 0.01 | 0.04 | 0.01 | -0.01 | 0.04 | 0.00 | 0.25 |
| 3 | 0.00 | 0.01 | 0.00 | -0.01 | 0.02 | | 0.01 | 0.02 | 0.05 | 0.02 | 0.02 | 0.01 | | 0.00 | 0.01 | 0.04 | 0.01 | -0.01 | 0.03 | 0.00 | 0.25 |
| 4 | 0.00 | 0.01 | 0.00 | -0.02 | 0.02 | | 0.01 | 0.02 | 0.06 | 0.02 | 0.03 | 0.01 | | 0.01 | 0.01 | 0.05 | 0.01 | -0.02 | 0.03 | 0.00 | 0.25 |
| 5 | 0.01 | 0.02 | -0.01 | -0.01 | 0.02 | | 0.02 | 0.02 | 0.05 | 0.03 | 0.03 | 0.01 | | 0.01 | 0.01 | 0.05 | 0.01 | -0.02 | 0.04 | 0.00 | 0.20 |
| 6 | 0.01 | 0.02 | -0.01 | 0.00 | 0.02 | | 0.02 | 0.02 | 0.04 | 0.02 | 0.03 | 0.01 | | 0.01 | 0.01 | 0.05 | 0.01 | -0.02 | 0.04 | 0.00 | 0.16 |
| 7 | 0.01 | 0.02 | -0.01 | 0.00 | 0.02 | | 0.02 | 0.02 | 0.04 | 0.02 | 0.03 | 0.01 | | 0.01 | 0.01 | 0.05 | 0.01 | -0.02 | 0.04 | 0.00 | 0.16 |
| 8 | 0.01 | 0.02 | -0.01 | -0.01 | 0.02 | | 0.02 | 0.02 | 0.04 | 0.02 | 0.03 | 0.01 | | 0.01 | 0.01 | 0.05 | 0.01 | -0.02 | 0.04 | 0.00 | 0.17 |
| 9 | 0.01 | 0.02 | -0.01 | -0.02 | 0.02 | | 0.02 | 0.02 | 0.04 | 0.02 | 0.02 | 0.01 | | 0.01 | 0.01 | 0.05 | 0.01 | -0.02 | 0.03 | 0.00 | 0.18 |
| 10 | 0.01 | 0.02 | -0.01 | -0.02 | 0.02 | | 0.02 | 0.02 | 0.04 | 0.02 | 0.02 | 0.01 | | 0.01 | 0.01 | 0.05 | 0.01 | -0.02 | 0.03 | 0.00 | 0.17 |
| 11 | 0.01 | 0.02 | -0.01 | -0.02 | 0.02 | | 0.02 | 0.02 | 0.05 | 0.02 | 0.02 | 0.01 | | 0.01 | 0.01 | 0.06 | 0.01 | -0.02 | 0.03 | 0.00 | 0.17 |
| 12 | 0.01 | 0.02 | -0.01 | -0.02 | 0.02 | | 0.02 | 0.02 | 0.05 | 0.03 | 0.03 | 0.01 | | 0.01 | 0.01 | 0.06 | 0.01 | -0.02 | 0.04 | 0.00 | 0.17 |
| 13 | 0.01 | 0.02 | -0.01 | -0.01 | 0.02 | | 0.02 | 0.02 | 0.05 | 0.03 | 0.03 | 0.01 | | 0.01 | 0.02 | 0.07 | 0.01 | -0.02 | 0.04 | 0.00 | 0.17 |
| 14 | 0.01 | 0.02 | -0.01 | -0.01 | 0.02 | | 0.02 | 0.02 | 0.05 | 0.03 | 0.03 | 0.01 | | 0.01 | 0.02 | 0.07 | 0.01 | -0.02 | 0.04 | 0.00 | 0.17 |
| 15 | 0.01 | 0.02 | -0.01 | -0.01 | 0.02 | | 0.02 | 0.02 | 0.05 | 0.03 | 0.03 | 0.01 | | 0.01 | 0.02 | 0.07 | 0.01 | -0.02 | 0.04 | 0.00 | 0.17 |
| 16 | 0.01 | 0.02 | -0.01 | -0.01 | 0.02 | | 0.02 | 0.02 | 0.05 | 0.03 | 0.03 | 0.01 | | 0.01 | 0.01 | 0.05 | 0.01 | -0.02 | 0.04 | 0.00 | 0.17 |
| 17 | 0.01 | 0.02 | 0.00 | -0.02 | 0.02 | | 0.02 | 0.02 | 0.04 | 0.03 | 0.01 | 0.01 | | 0.01 | 0.01 | 0.04 | 0.01 | -0.02 | 0.03 | 0.00 | 0.14 |
| 18 | 0.01 | 0.02 | 0.00 | -0.04 | 0.02 | | 0.02 | 0.02 | 0.04 | 0.03 | 0.01 | 0.01 | | 0.01 | 0.01 | 0.04 | 0.01 | -0.02 | 0.03 | 0.00 | 0.13 |
| 19 | 0.01 | 0.02 | 0.00 | -0.04 | 0.02 | | 0.02 | 0.02 | 0.04 | 0.03 | 0.02 | 0.01 | | 0.00 | 0.01 | 0.04 | 0.01 | -0.02 | 0.03 | 0.00 | 0.13 |
| 20 | 0.00 | 0.01 | -0.01 | -0.10 | 0.02 | | 0.02 | 0.02 | 0.02 | 0.03 | 0.02 | 0.00 | | 0.00 | -0.01 | 0.05 | 0.01 | -0.02 | 0.01 | 0.00 | 0.11 |
| 21 | 0.00 | 0.01 | -0.01 | -0.11 | 0.02 | | 0.02 | 0.02 | 0.02 | 0.03 | 0.02 | 0.00 | | 0.00 | -0.01 | 0.05 | 0.01 | -0.02 | 0.01 | 0.00 | 0.11 |
| 22 | 0.00 | 0.01 | -0.01 | -0.23 | 0.02 | | 0.02 | 0.02 | 0.02 | 0.03 | 0.00 | 0.00 | | 0.01 | -0.01 | 0.04 | 0.01 | -0.02 | 0.01 | 0.00 | 0.10 |
| 23 | 0.00 | 0.01 | -0.01 | -0.78 | 0.02 | | 0.01 | 0.02 | 0.02 | 0.03 | -0.05 | 0.00 | | 0.00 | -0.01 | 0.05 | 0.01 | -0.01 | 0.01 | 0.01 | 0.09 |
| 24 | 0.00 | 0.01 | -0.01 | -0.10 | 0.01 | | 0.01 | 0.02 | 0.02 | 0.02 | NA | 0.00 | | 0.00 | -0.01 | 0.05 | 0.01 | -0.01 | 0.01 | 0.01 | 0.08 |
| 25 | 0.00 | 0.01 | -0.01 | -0.10 | 0.01 | | 0.01 | 0.02 | 0.02 | 0.02 | NA | 0.00 | | 0.00 | -0.01 | 0.05 | 0.01 | -0.01 | 0.01 | 0.01 | 0.08 |
| 26 | -0.01 | 0.01 | -0.02 | -0.11 | 0.01 | | 0.01 | 0.02 | 0.02 | 0.02 | NA | 0.00 | | 0.00 | -0.01 | 0.05 | 0.01 | -0.01 | 0.01 | 0.01 | 0.09 |
| 27 | -0.01 | 0.01 | -0.02 | -0.10 | 0.01 | | 0.01 | 0.02 | 0.02 | 0.02 | NA | 0.00 | | 0.00 | -0.01 | 0.05 | 0.01 | -0.01 | 0.01 | 0.01 | 0.09 |
| 28 | -0.01 | 0.01 | -0.02 | -0.10 | 0.02 | | 0.01 | 0.02 | 0.02 | 0.02 | -0.10 | 0.00 | | 0.00 | -0.01 | 0.04 | 0.01 | -0.01 | 0.01 | 0.01 | 0.09 |
| 29 | -0.01 | 0.01 | -0.02 | -0.11 | 0.02 | | 0.01 | 0.02 | 0.02 | 0.02 | -0.09 | 0.00 | | 0.01 | -0.01 | 0.04 | 0.01 | -0.01 | 0.01 | 0.01 | 0.08 |
| 30 | -0.01 | 0.01 | -0.02 | -0.11 | 0.02 | | 0.01 | 0.02 | 0.02 | 0.02 | -0.09 | 0.00 | | 0.01 | -0.01 | 0.00 | 0.01 | -0.01 | 0.01 | 0.01 | 0.08 |

**Table S10**. R²-values from generalized linear mixed effects model to fit the activity density of the different ground-dwelling arthropod taxa/ plant cover in relation to the days before rainfall summed up for 30 days before the start of the pitfall trapping/ vegetation assessment.

|  | Herbivores | | | | | Omnivores | | | | | | Predators | | | | | | Detriti-vores | Ants (For-mici-dae) | Plant cover |
| --- | --- | --- | --- | --- | --- | --- | --- | --- | --- | --- | --- | --- | --- | --- | --- | --- | --- | --- | --- | --- |
| Day | Herbi-vores | Cica-das (Cica-dina) | Short-horned grass-hopper (Caeli-fera). | Aphids and scale insects (Stern-orrhyncha) | Omni-vores | | Beetle(Cole-optera) | Cock-roach-es (Blattodea) | True bugs (Heter-optera) | Long-horned grass-hopper(Ensifera) | Pso-cids or book-lice (Pso-cop-tera) | Preda-tors | Spider (Ara-neae) | Ticks and mites (Aca-rina) | Centi-pedes (Chilo-poda) | Scor-pions (Scor-piones) | False scor-pions (Pseudo-scor-piones) | Spring-tails (Coll-embo-la) |  |  |
| 0 | 0.09 | -0.04 | 0.16 | 0.00 | 0.05 | | 0.02 | 0.02 | -0.36 | 0.08 | 0.01 | 0.01 | 0.01 | 0.01 | 0.23 | 0.01 | 0.08 | 0.04 | -0.01 | 0.64 |
| 1 | 0.05 | -0.21 | 0.16 | 0.00 | 0.04 | | 0.02 | 0.02 | -0.59 | 0.07 | 0.00 | 0.01 | 0.00 | 0.01 | 0.24 | 0.01 | 0.08 | 0.03 | -0.01 | 0.62 |
| 2 | 0.04 | -0.19 | 0.15 | 0.00 | 0.03 | | 0.02 | 0.02 | -2.09 | 0.05 | 0.00 | 0.01 | 0.00 | 0.01 | 0.24 | 0.01 | 0.08 | 0.03 | 0.00 | 0.62 |
| 3 | 0.01 | -0.18 | 0.15 | 0.00 | 0.03 | | 0.02 | 0.03 | -2.48 | 0.05 | 0.00 | 0.01 | 0.01 | 0.01 | 0.24 | 0.01 | 0.10 | 0.03 | 0.00 | 0.63 |
| 4 | 0.03 | 0.08 | 0.16 | 0.00 | 0.04 | | 0.02 | 0.03 | -0.03 | 0.10 | 0.01 | 0.01 | 0.03 | 0.00 | 0.14 | 0.01 | 0.10 | 0.03 | -0.01 | 0.56 |
| 5 | 0.09 | 0.17 | 0.16 | 0.00 | 0.00 | | 0.02 | 0.02 | -0.07 | 0.07 | 0.02 | 0.02 | 0.06 | 0.01 | -0.01 | 0.02 | 0.10 | 0.01 | 0.00 | 0.30 |
| 6 | 0.10 | 0.16 | 0.16 | 0.00 | -0.02 | | 0.02 | 0.02 | -0.14 | 0.06 | 0.01 | 0.02 | 0.06 | 0.01 | -0.05 | 0.02 | 0.09 | 0.01 | 0.00 | 0.22 |
| 7 | 0.10 | 0.16 | 0.16 | 0.00 | -0.02 | | 0.02 | 0.02 | -0.14 | 0.06 | 0.01 | 0.02 | 0.06 | 0.01 | -0.05 | 0.02 | 0.10 | 0.01 | 0.00 | 0.22 |
| 8 | 0.09 | 0.16 | 0.16 | 0.00 | 0.01 | | 0.02 | 0.02 | -0.03 | 0.07 | 0.01 | 0.02 | 0.06 | 0.01 | -0.01 | 0.02 | 0.10 | 0.01 | 0.00 | 0.24 |
| 9 | 0.07 | 0.12 | 0.16 | 0.00 | 0.04 | | 0.03 | 0.02 | 0.07 | 0.09 | 0.01 | 0.01 | 0.04 | 0.01 | 0.06 | 0.01 | 0.10 | 0.02 | 0.00 | 0.28 |
| 10 | 0.07 | 0.11 | 0.16 | 0.01 | 0.05 | | 0.03 | 0.02 | 0.09 | 0.08 | 0.01 | 0.01 | 0.04 | 0.01 | 0.09 | 0.01 | 0.10 | 0.02 | 0.01 | 0.28 |
| 11 | 0.07 | 0.12 | 0.16 | 0.01 | 0.06 | | 0.03 | 0.02 | 0.10 | 0.08 | 0.01 | 0.01 | 0.03 | 0.01 | 0.12 | 0.01 | 0.10 | 0.02 | 0.01 | 0.28 |
| 12 | 0.10 | 0.17 | 0.16 | 0.00 | 0.03 | | 0.03 | 0.02 | 0.00 | 0.05 | 0.02 | 0.02 | 0.04 | 0.01 | 0.05 | 0.01 | 0.10 | 0.01 | 0.01 | 0.21 |
| 13 | 0.11 | 0.20 | 0.16 | 0.01 | 0.02 | | 0.03 | 0.01 | -0.04 | 0.04 | 0.02 | 0.02 | 0.04 | 0.01 | 0.05 | 0.01 | 0.12 | 0.02 | 0.01 | 0.20 |
| 14 | 0.11 | 0.20 | 0.16 | 0.01 | 0.02 | | 0.03 | 0.02 | -0.04 | 0.04 | 0.02 | 0.02 | 0.04 | 0.01 | 0.05 | 0.01 | 0.12 | 0.02 | 0.01 | 0.21 |
| 15 | 0.11 | 0.20 | 0.16 | 0.01 | 0.03 | | 0.03 | 0.02 | -0.02 | 0.05 | 0.01 | 0.02 | 0.04 | 0.01 | 0.05 | 0.01 | 0.12 | 0.02 | 0.01 | 0.20 |
| 16 | 0.11 | 0.19 | 0.16 | 0.01 | 0.04 | | 0.03 | 0.02 | 0.02 | 0.05 | 0.01 | 0.02 | 0.03 | 0.01 | 0.03 | 0.01 | 0.11 | 0.02 | 0.01 | 0.22 |
| 17 | 0.13 | 0.17 | 0.16 | 0.01 | 0.16 | | 0.10 | 0.01 | -0.25 | 0.13 | 0.00 | 0.01 | 0.02 | 0.01 | 0.03 | 0.01 | 0.11 | 0.00 | 0.01 | 0.19 |
| 18 | 0.11 | 0.14 | 0.16 | 0.00 | 0.16 | | 0.11 | 0.01 | -0.36 | 0.14 | 0.00 | 0.01 | 0.02 | 0.00 | 0.03 | 0.01 | 0.11 | 0.00 | 0.01 | 0.17 |
| 19 | 0.11 | 0.14 | 0.16 | 0.01 | 0.16 | | 0.11 | 0.01 | -0.45 | 0.13 | 0.00 | 0.01 | 0.01 | 0.00 | 0.03 | 0.01 | 0.10 | -0.01 | 0.01 | 0.16 |
| 20 | 0.01 | -0.15 | 0.17 | 0.02 | 0.13 | | 0.09 | 0.01 | 0.00 | 0.12 | 0.00 | 0.00 | 0.01 | 0.01 | 0.03 | 0.01 | 0.10 | 0.01 | 0.01 | 0.11 |
| 21 | 0.01 | -0.15 | 0.17 | 0.02 | 0.14 | | 0.09 | 0.01 | -0.01 | 0.12 | 0.00 | 0.00 | 0.01 | 0.01 | 0.04 | 0.01 | 0.10 | 0.01 | 0.01 | 0.10 |
| 22 | -0.01 | -0.21 | 0.16 | 0.04 | 0.16 | | 0.12 | 0.02 | 0.00 | 0.13 | 0.00 | 0.00 | 0.01 | 0.01 | 0.03 | 0.01 | 0.11 | 0.01 | 0.03 | 0.08 |
| 23 | -0.05 | -0.41 | 0.16 | 0.03 | 0.14 | | 0.08 | 0.02 | 0.01 | 0.14 | 0.02 | 0.00 | 0.01 | 0.01 | 0.15 | 0.01 | 0.11 | 0.01 | 0.05 | 0.09 |
| 24 | -0.06 | -0.45 | 0.16 | 0.03 | 0.13 | | 0.08 | 0.02 | 0.01 | 0.13 | NA | 0.00 | 0.01 | 0.01 | 0.14 | 0.01 | 0.10 | 0.01 | 0.06 | 0.08 |
| 25 | -0.06 | -0.45 | 0.16 | 0.03 | 0.13 | | 0.08 | 0.02 | 0.01 | 0.13 | NA | 0.00 | 0.01 | 0.01 | 0.13 | 0.01 | 0.10 | 0.01 | 0.06 | 0.08 |
| 26 | -0.07 | -0.56 | 0.17 | 0.03 | 0.08 | | 0.04 | 0.02 | 0.02 | 0.08 | NA | 0.00 | 0.01 | 0.01 | 0.14 | 0.01 | 0.10 | 0.01 | 0.06 | 0.08 |
| 27 | -0.07 | -0.54 | 0.17 | 0.03 | 0.08 | | 0.04 | 0.02 | 0.02 | 0.07 | NA | 0.00 | 0.01 | 0.01 | 0.13 | 0.01 | 0.10 | 0.01 | 0.07 | 0.08 |
| 28 | -0.07 | -0.53 | 0.14 | 0.03 | 0.07 | | 0.04 | 0.02 | 0.02 | 0.06 | 0.01 | 0.00 | 0.01 | 0.01 | 0.11 | 0.01 | 0.10 | 0.01 | 0.08 | 0.07 |
| 29 | -0.07 | -0.51 | 0.14 | 0.03 | 0.07 | | 0.04 | 0.02 | 0.02 | 0.05 | 0.00 | 0.00 | 0.01 | 0.01 | 0.09 | 0.01 | 0.10 | 0.01 | 0.08 | 0.07 |
| 30 | -0.07 | -0.49 | 0.14 | 0.03 | 0.06 | | 0.04 | 0.02 | 0.01 | 0.04 | 0.00 | 0.00 | 0.02 | 0.01 | -0.07 | 0.01 | 0.10 | 0.01 | 0.08 | 0.05 |
